# Immune and metabolic effects of African heritage diets versus Western diets in men: a randomized controlled trial

**DOI:** 10.1038/s41591-025-03602-0

**Published:** 2025-04-03

**Authors:** Godfrey S. Temba, Tal Pecht, Vesla I. Kullaya, Nadira Vadaq, Mary V. Mosha, Thomas Ulas, Sneha Kanungo, Liesbeth van Emst, Lorenzo Bonaguro, Jonas Schulte-Schrepping, Elias Mafuru, Paolo Lionetti, Musa M. Mhlanga, Andre J. van der Ven, Duccio Cavalieri, Leo A. B. Joosten, Reginald A. Kavishe, Blandina T. Mmbaga, Joachim L. Schultze, Mihai G. Netea, Quirijn de Mast

**Affiliations:** 1 Department of Medical Biochemistry and Molecular Biology, KCMC University, Moshi, United Republic of Tanzania; 2 Department of Internal Medicine, Radboudumc Research Institute for Medical Innovation (RIMI), Radboud University Medical Center, Nijmegen, the Netherlands; 3 Department for Genomics and Immunoregulation, Life & Medical Sciences (LIMES) Institute, University of Bonn, Bonn, Germany; 4 Systems Medicine, German Center for Neurodegenerative Diseases (DZNE), Bonn, Germany; 5 Institute of Clinical Chemistry and Clinical Pharmacology, University Hospital Bonn, Bonn, Germany; 6 Kilimanjaro Clinical Research Institute, Kilimanjaro Christian Medical Center, Moshi, United Republic of Tanzania; 7 School of Public Health, KCMC University, Moshi, United Republic of Tanzania; 8 PRECISE Platform for Single Cell Genomics and Epigenomics, German Center for Neurodegenerative Diseases (DZNE) and University of Bonn, Bonn, Germany; 9 Department NEUROFARBA, University of Florence, Florence, Italy; 10 Gastroenterology and Nutrition Unit, Meyer Children’s Hospital IRCCS, Florence, Italy; 11 Department of Cell Biology, Faculty of Science, Radboud University, Nijmegen, the Netherlands; 12 Department of Human Genetics, Radboud University Medical Center, Nijmegen, the Netherlands; 13 Department of Biology, University of Florence, Florence, Italy; 14 Department of Medical Genetics, Iuliu Hatieganu University of Medicine and Pharmacy, Cluj-Napoca, Romania; 15 Department of Immunology and Metabolism, Life & Medical Sciences (LIMES) Institute, University of Bonn, Bonn, Germany

**Keywords:** Translational immunology, Translational research

## Abstract

African heritage diets are increasingly being replaced by Western-style dietary patterns because of urbanization, economic development, increased access to processed foods, globalization and changing social norms. The health consequences of this nutrition transition are not well understood. We conducted a randomized controlled trial in the Kilimanjaro region in Northern Tanzania to investigate the immune and metabolic effects of switching between Kilimanjaro heritage-style and Western-style diets for 2 weeks and consuming a traditional fermented banana beverage (‘Mbege’) for 1 week. Seventy-seven young and healthy volunteers assigned male at birth, some living in urban areas and some living in rural areas, were recruited in the trial. Primary outcomes were changes in the immune and metabolic profile before and after the intervention and at the 4-week follow-up. The switch from heritage-style to Western-style diet affected different metabolic pathways associated with noncommunicable diseases and promoted a pro-inflammatory state with impaired whole-blood cytokine responses to microbial stimulation. In contrast, the switch from Western-style to heritage-style diet or consuming the fermented beverage had a largely anti-inflammatory effect. Some of the observed changes in the immune and metabolic profiles persisted at the follow-up, suggesting a sustained impact from the short-term intervention. These findings show the metabolic and immune effects of dietary transitions and the consumption of fermented beverages, underscoring the importance of preserving indigenous dietary practices to mitigate noncommunicable disease risk factors in sub-Saharan Africa. ISRCTN trial registration: ISRCTN15619939.

## Abstract (web summary)

A randomized crossover trial showed that transition to a Western diet resulted in pro-inflammatory changes, whereas transition to an African heritage diet rich in fruits and vegetables, legumes and fermented products led to opposite effects.

## Main

Sub-Saharan Africa faces a surge in noncommunicable diseases (NCDs), including cardiovascular disease (CVD), diabetes and chronic inflammatory conditions^1^, straining health systems. Urban adult mortality rates now exceed rural rates^2^, largely because of lifestyle changes, including the adoption of calorie-dense Western diets (WDs). Africa has a rich diversity of heritage diets, often rich in vegetables, fruits, legumes, grains and fermented products. Plant-based diets and fermented foods are increasingly recognized for their beneficial immune and metabolic effects^3–5^. In contrast, Western-style diets, high in calorie-dense and processed foods, drive metabolic dysfunction, systemic inflammation and long-term immune cell reprogramming, a phenomenon called ‘maladaptive trained immunity’^6,7^, which are key drivers of NCDs^8^.

African populations are severely underrepresented in nutritional and immunological research, with limited data available on the immune and metabolic impact of African heritage diets (AHDs) compared to well-studied diets such as the Mediterranean or Japanese diet. In a previous cross-sectional study of healthy Tanzanian individuals from the Kilimanjaro region, we found that dietary patterns were associated with cytokine responses, plasma proteome and coagulation markers^9–11^. Urban residents, with greater exposure to WD environments, exhibited a stronger pro-inflammatory profile than those living in rural areas. Most rural dwellers in the study were from the Chagga tribe, whose heritage diet is predominantly plant-based, rich in fiber and polyphenols, and includes ‘Mbege’, a fermented banana beverage made with finger millet^12^. Fermented foods, rich in bioactive microbes, have garnered renewed interest for their health benefits, yet they are becoming increasingly scarce in modern diets because of industrial food processing^13,14^.

Cross-sectional observational studies are limited in establishing causal relationships and controlling confounders. Therefore, we performed an open-label, randomized controlled trial to evaluate the short-term and long-term immunometabolic effects of a short dietary intervention in young Tanzanian men from rural and urban backgrounds. The study investigated the effects of switching from a Western-style diet to a Kilimanjaro heritage diet and vice versa, and the effects of consuming the fermented beverage ‘Mbege’. Changes in circulating leukocyte profiles, the inflammatory and cardiometabolic proteome, whole-blood cytokine responses and transcriptome, and plasma metabolome were assessed at baseline, immediately after the intervention and 4 weeks later.

## Results

### Participant characteristics and dietary recall

Seventy-seven healthy adult men (median age = 25.6 years; interquartile range (IQR) = 21–27.2) participated in an open-label, short dietary intervention with 4-week follow-up to assess its longer-term immunometabolic effects. Participants were recruited from 22 April 2021 to 3 July 2021 with the final sampling on 9 August 2021. Baseline characteristics are summarized in Extended Data Table 1. Participants with a body mass index (BMI) of 18–25.9 were eligible for inclusion; however, four participants with a BMI > 26 were inadvertently included. Dietary habits were assessed using three nonconsecutive 24-h recalls, including a festival or weekend day, and are summarized in Supplementary Table 1.

**Fig. 1:**
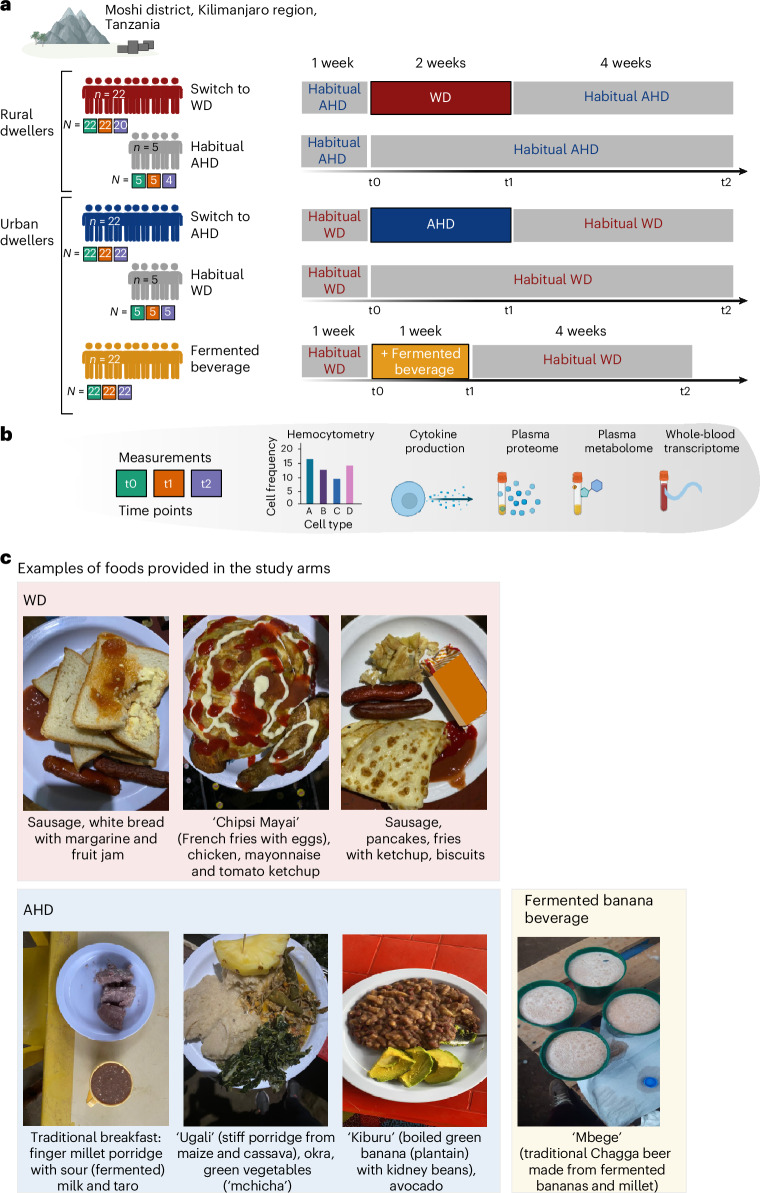
Schematic depiction of the trial. **a**, The study was conducted at the Moshi district, Kilimanjaro region, Tanzania. The illustration describes the dietary interventions carried out in this study. After one participant withdrew from the study before the end of the intervention, the first group included young rural-living men (*n* = 22) who entered the intervention, whose habitual diet mainly consisted of a Kilimanjaro AHD and who changed for 2 weeks to a WD (top). The second group consisted of urban-living young men (*n* = 22) whose habitual diet mainly consisted of a WD, and who were switched for 2 weeks to a plant-based, fiber-rich heritage-style diet (middle). In both groups, five controls who remained on their habitual diet were also enrolled. The third group consisted of young men (*n* = 22) whose diet remained unchanged from their habitual WD, but who were supplemented with consumption of a locally brewed fermented banana beverage (Mbege), approximately 1 l per day for 1 week. Below each group, the number of participants sampled at each of the time points (baseline, t0; after the intervention, t1; and at the follow-up, t2), are presented in green, orange and purple filled squares, respectively. **b**, In all study arms, samples for hemocytometry, whole-blood cytokine responses and RNA sequencing (RNA-seq), as well as plasma proteome and metabolome, were collected at three time points: baseline (t0); on completion of the intervention (after the intervention) (t1) (at week 2 for the dietary intervention and week 1 for the banana beverage intervention); and 4 weeks after completion of the intervention (t2) (‘follow-up’). **c**, Images of example foods and the fermented beverage provided in the study arms. Schematic in **a** created using BioRender.com.

The trial, depicted in Fig. 1a, consisted of three arms: (1) men living in rural areas (*n* = 23) who habitually consumed a Kilimanjaro heritage-style diet and switched to a Western-style diet for 2 weeks; (2) men living in urban areas (*n* = 22) who habitually consumed a Western-style diet and switched to a heritage-style diet for 2 weeks; and (3) men who habitually consumed a Western-style diet (*n* = 22) and who consumed Mbege, a traditional fermented banana beverage, for 1 week. Five participants who maintained their habitual diets were included in the first two arms as controls to assess diet-independent effects (Supplementary Tables 2 and 3). Blood samples were collected at three time points: baseline; after the intervention (immediately after the 2-week dietary intervention or 1-week fermented beverage intervention); and at the follow-up (4 weeks after the intervention).

**Extended Data Fig. 1:**
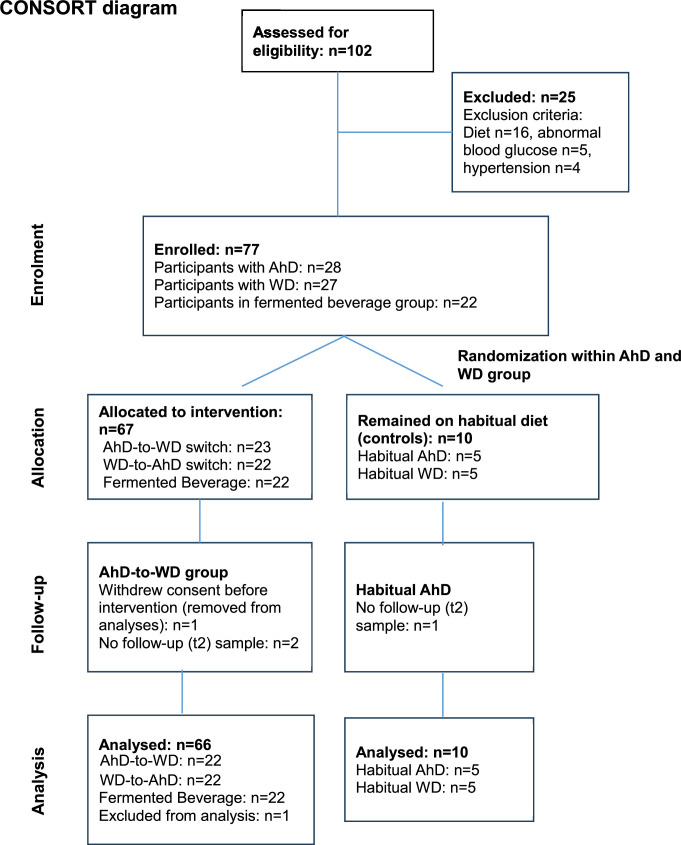
CONSORT diagram. Flow diagram illustrating the enrollment, allocation, follow-up, and analysis of participants.

In the group of participants switching from AHD to WD, one participant withdrew before the start of the intervention and was excluded from the analyses, while two participants in this arm and one control lacked follow-up samples (CONSORT diagram in Extended Data Fig. 1). Outcomes were assessed based on sample availability and quality control (Fig. 1a,b and Supplementary Table 4), and were adjusted according to age, BMI and activity level (Supplementary Table 5).

Meals were provided three times daily in the Uru Shimbwe Juu village (AHD to WD switch) or Moshi town (WD to AHD switch). Examples and a detailed description of switched diets are shown in Fig. 1c and Extended Data Table 2. Weight monitoring showed a notable increase in the group who switched to a WD (median = 2.6 kg, IQR = 2.0–3.4 kg, *P* < 0.001; Mann–Whitney *U*-test).

### Multiomics overview of immunometabolic changes

**Extended Data Fig. 2:**
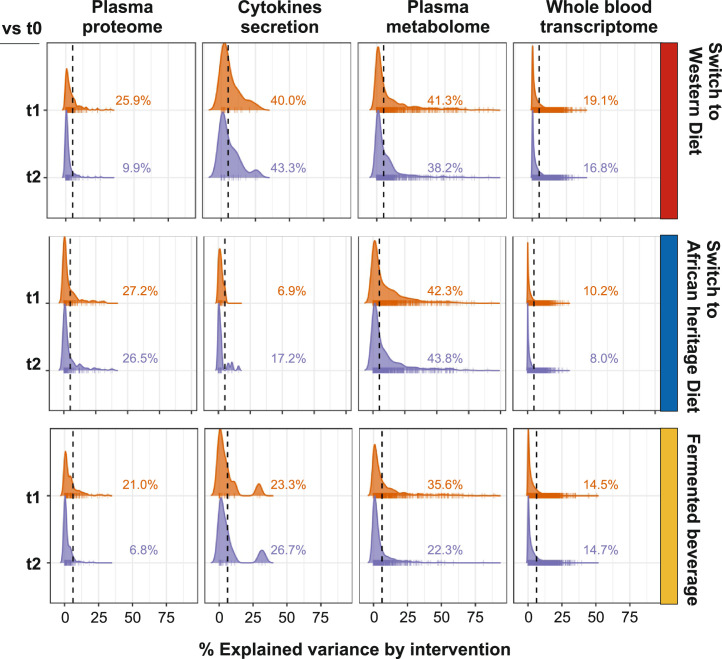
Percentage of the explained variation attributed to the diet intervention for multiplex / omics data layer. Histograms of the percentage of explained variance by dietary intervention in the switch from African heritage diet (AHD) to Western diet (Switch to WD), switch from Western to African heritage diet (Switch to AHD), consumption of the fermented beverage (top, middle and bottom, respectively). Variance partition analysis was performed considering the change in features in each omics layer between post-intervention (t1, orange) or follow-up (t2, purple) vs baseline (t0) measurements. Age, BMI and physical activity levels at baseline were included as additional fixed variables and individual ID as random effect. The percentage of features in each data layer that intervention contributed to more than 5% in its variance is marked in each plot.

To assess the impact of the intervention on the variance in plasma proteome, cytokine production, plasma metabolome and whole-blood transcriptome, which are primary outcomes of the trial, we conducted variance partition analysis, using the intervention time points as the primary variable, with age, BMI and physical activity as covariates, and participant ID as a random effect (Extended Data Fig. 2). Each intervention induced distinct immune and metabolic adaptations both in the short (after the intervention versus baseline) and long (follow-up versus baseline) term. Switching from an AHD to a WD accounted for over 5% of variance in plasma proteome (25.9%), cytokine production (40%), plasma metabolome (41.3%) and whole-blood transcriptome (19.1%) features, respectively. These effects largely persisted at the follow-up except for plasma proteome variance, which reduced to 9.9%. Conversely, switching from a WD to an AHD mainly influenced plasma proteome and metabolome, explaining over 5% of variance in 27% and 42% of features, respectively. Fermented beverage consumption explained over 5% of variance in plasma proteome (21%), cytokine production (23.3%), plasma metabolome (35.6%) and whole-blood transcriptome (14.5%) features, with sustained variation primarily in cytokine production and whole-blood transcriptome.

### Plasma inflammatory and cardiometabolic proteomes

Next, we analyzed changes in circulating proteins related to inflammation and metabolism, a prespecified primary outcome, using the 92-plex Olink inflammatory and cardiometabolic panels (proximity extension assays, Olink Proteomics AB)^15^. Relative protein concentrations were analyzed for 162 proteins (89 cardiometabolic, 73 inflammatory; Supplementary Table 6) after excluding proteins with values below the detection limits in more than 25% of samples. Differentially abundant proteins (DAPs) were identified using a linear mixed model (LMM) analysis and annotated to biological processes according to the Human Protein Atlas (v.23.0). Analysis focused on proteins that the time point variable explained over 5% of the variance (Methods and Supplementary Table 6).

**Fig. 2:**
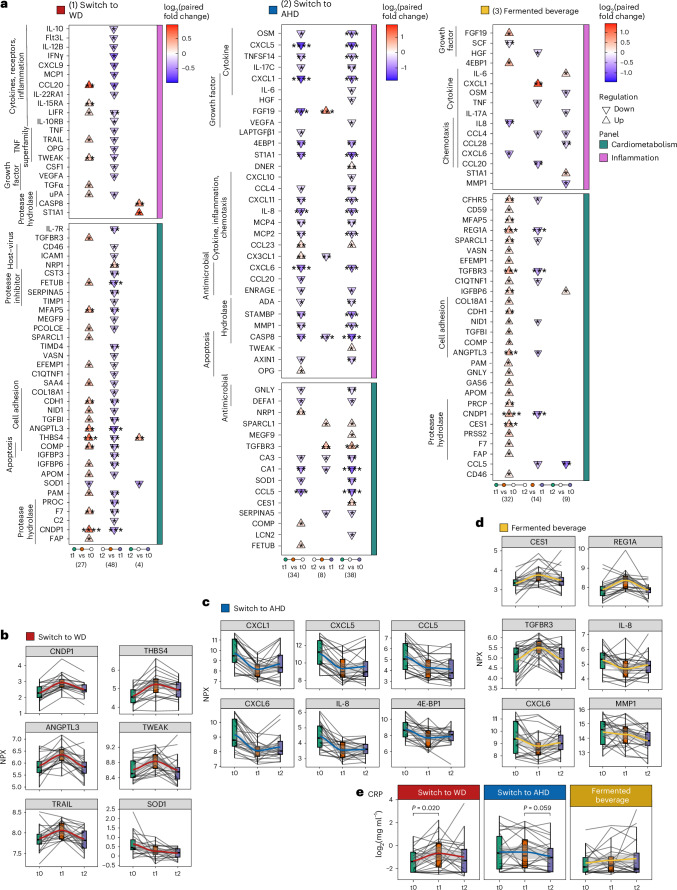
Differentially abundant inflammatory and cardiometabolic proteins. Targeted plasma proteomics was conducted using the inflammatory and cardiometabolic Olink panel. Plasma CRP concentrations were measured using ELISA. Samples were obtained at baseline (t0), after the intervention (t1, week 2 for the dietary intervention and 1 week for the fermented beverage intervention) and 4 weeks later (t2, follow-up). Statistical analysis was carried out for participants who completed the full study, that is *n* = 20, 22 and 22 in the WD, AHD and fermented beverage intervention groups, respectively. Statistical analysis was carried out using an LMM for each comparison with a two-sided hypothesis test. Fixed effects included time point, baseline BMI, age and physical activity level; participant ID was included as a random effect. **a**, Triangle plots present DAPs in the (I) WD, (II) AHD and (III) fermented beverage intervention groups. The direction of the arrow (up or down) indicates the direction of change, comparing late versus early time points in each comparison as listed at the bottom of the plot, with the color fill depicting the log_2_ paired fold change value for each comparison. **b**–**e**, Combined box and line plots with the group trend line indicating the levels of measured parameters at the three time points. Lines are grouped per participant. Protein concentrations are presented as Normalized Protein eXpression (NPX) values for Olink measures (switch to WD (**b**), switch to AHD (**c**) and fermented beverage (**d**)) or log_2_-transformed CRP levels (mg ml^−1^, **e**). In all box plots, the line defines the median level, the hinges depict the 25th and 75th centiles and the whiskers extend to ±1.5 times the IQR. The asterisks indicate the significance level based on Benjamini–Hochberg-adjusted *P* values (*P < 0.05, **P < 0.01, ***P < 0.001, ****P < 0.0001).

**Extended Data Fig. 3:**
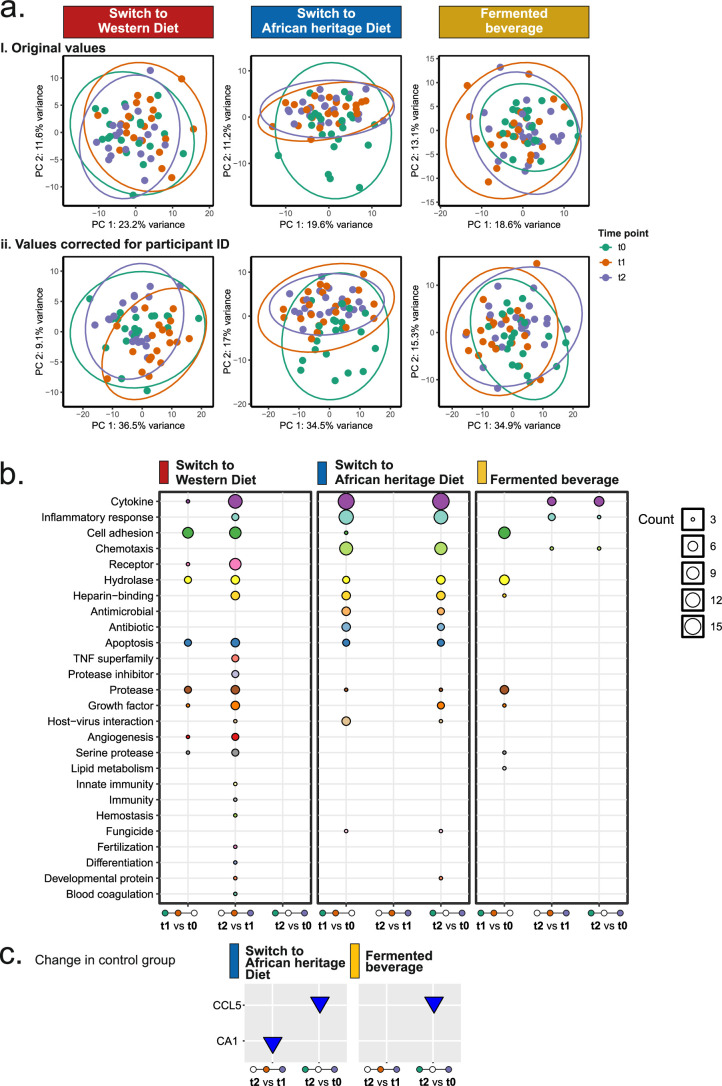
Principal component analysis (PCA) of plasma proteome and pathway annotation. Targeted plasma proteomics was conducted using the inflammatory and cardiometabolic Olink panel (Olink® Proteomics AB). Samples from N = 20, 22 and 22 individuals in the switch from African heritage to Western diet (Switch to WD), switch from Western to African heritage diet (Switch to AHD) and the fermented beverage intervention arms were obtained at baseline (t0), post-intervention (t1), and four weeks follow-up (t2). **a**- PCA was calculated on (i) scaled original NPX values, or (ii) scaled values corrected for participant ID. Dot plots depict the principal component 1 (PC1) and PC2, with color indicating the measurement time point (t0, t1 and t2 in green, orange and purple) and geometric ellipses drawn at 95% confidence for each time point. **b**- Dot plot showing the biological terms (derived from the Human Protein Atlas) for the differential abundant proteins in each intervention arm and time point comparison, with size indicating the number of proteins related to each term.

Principal component analysis (PCA) of participant-corrected values revealed distinct shifts, which were more pronounced in participants switching from a WD to an AHD (Extended Data Fig. 3a). Participants switching from an AHD to a WD exhibited a significant increase in 26 proteins (19 cardiometabolic and seven inflammation panel) and a decrease in one protein after the intervention compared to baseline (Fig. 2a,b, Supplementary Fig. 1 and Supplementary Table 7). Among the most upregulated proteins were CNDP1, THBS4 (implicated in atherogenesis^16^), ANGPTL3 and TWEAK (Fig. 2a,b). Key biological processes linked to DAPs included ‘cell adhesion’ (for example, THBS2, NID1, CDH1), ‘hydrolase activity’ (for example, CNDP1, PAM), ‘apoptosis’ (for example, TWEAK, TRAIL, TGFB1) and ‘protease activity’ (for example, ANGPTL3, uPA, F7) (Extended Data Fig. 3b), suggesting early pathological adaptations to a WD, characterized by systemic inflammation and metabolic dysregulation.

In contrast, participants switching from a WD to an AHD exhibited a significant reduction in 28 proteins (22 inflammation, 6 cardiometabolic) and an increase in 6 proteins after the intervention compared to baseline (Fig. 2a,c, Supplementary Fig. 2 and Supplementary Table 7). These DAPs were linked to ‘cytokines,’ ‘inflammatory response,’ ‘chemotaxis’ and ‘host–virus interactions’ (Extended Data Fig. 3b). At the follow-up, the changes had partially reversed; however, several proteins, including chemokines (CXCL1, CXCL5, CXCL6, CXCL8, CXCL10, CXCL11, CCL4, CCL5, MCP2, MCP4), cytokines (IL-6, OSM, IL-17C, TNFSF14) and others (CASP8, MMP1, CA1, CA3, STAMBP, ST1A1, 4E-BP), remained significantly decreased compared to baseline. CASP8 and ST1A1 exhibited changes opposite to those in the AHD to WD group. In the control group, most DAPs remained unchanged, except for reduced values of CA1 (follow-up versus after the intervention) and CCL5 (follow-up versus baseline) (Extended Data Fig. 3c and Supplementary Table 7).

Consumption of the fermented beverage resulted in a more diverse pattern of protein changes, with plasma concentrations of 29 proteins (27 cardiometabolic, two inflammatory) increasing and three inflammatory proteins decreasing after the intervention compared to baseline (Fig. 2a,d, Supplementary Fig. 3 and Supplementary Table 7). At the follow-up time point, cardiometabolic proteins returned to baseline but inflammatory markers, including CC chemokines (CCL5, CCL4, CCL28), MMP1, IL-17A and OSM, remained lower than baseline. This is consistent with Wastyk et al.^5^, who observed reductions in inflammatory markers with a highly fermented food diet. In the control group, most DAPs remained unchanged, except for a decrease in CCL5 (follow-up versus baseline; Extended Data Fig. 3c and Supplementary Table 7).

To confirm the inflammatory effects, plasma C-reactive protein (CRP) concentrations were measured using a high-sensitivity enzyme-linked immunosorbent assay (ELISA). Samples with values below the detection threshold (24.4% of samples) were assigned the limit of the detection value (0.2 mg l^−1^; Fig. 2e). Using an LMM analysis as for the Olink data, a significant increase in plasma CRP concentrations was observed in participants switching to a WD (after the intervention versus baseline, *P* = 0.020), while a trend toward reduced CRP concentrations was observed in participants switching to an AHD (follow-up versus after the intervention, *P* = 0.059), with no significant changes in the fermented beverage group.

Overall, switching to a Kilimanjaro heritage-style diet resulted in sustained reductions in inflammatory and metabolic plasma proteins. Conversely, switching to a WD increased metabolic proteins and, to a lesser extent, inflammatory proteins, with minimal lasting effects. Fermented beverage consumption induced a rapid shift in cardiometabolic proteins and a sustained reduction in inflammatory proteins.

### Cytokine production capacity

Subsequently, we analyzed the effect of the interventions on cytokine production capacity, a prespecified primary outcome. Whole blood was incubated with *Candida albicans*, *Escherichia coli* lipopolysaccharide (LPS), *Mycobacterium tuberculosis* (MTB), *Salmonella enteritidis*, *Staphylococcus aureus* and the TLR3 agonist polyinosinic:polycytidylic acid (poly(I:C)) for 48 h. Cytokines (tumor necrosis factor (TNF), interleukin-6 (IL-6), IL-1β, IL-10, interferon-y (IFNγ)) were measured in supernatants using ELISAs, producing 30 cytokine response measures.

**Fig. 3:**
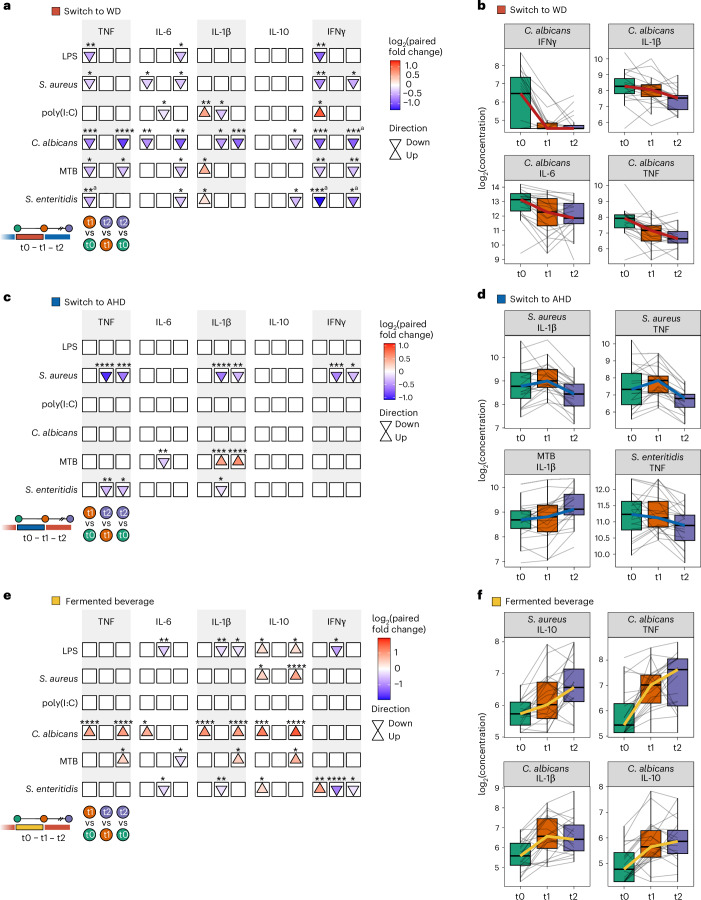
Changes in whole-blood cytokine production to stimulation with several microbial stimuli. Whole blood collected at baseline (t0), after the intervention (t1: week 2 for the dietary intervention group and week 1 for the banana beverage intervention group) and 4 weeks later (t2, follow-up), was subjected to 48-h ex vivo stimulation using the following stimuli: *C. albicans* hyphae (1 × 10^7^ ml^−1^), *E. coli* LPS (10 ng ml^−1^), MTB (5 µg ml^−1^), *S. enteritidis* (1 × 10^6^ ml^−1^), *S. aureus* (1 × 10^6^ ml^−1^) and the TLR3 agonist poly(I:C) (50 µg ml^−1^). Cytokine concentrations were measured in the supernatants at three time points for participants in the WD (*n* = 21), AHD (*n* = 22) and fermented beverage (*n* = 22) study arms using ELISA. Statistical analysis was carried out using an LMM for each comparison with a two-sided hypothesis test. Fixed effects included time point, baseline BMI, age and physical activity level, while participant ID was included as a random effect. **a**,**c**,**e**, Arrow plots depicting statistical comparisons between time points for switch to WD (**a**), switch to AHD (**c**) and fermented beverage (**e**): after the intervention (t1) versus baseline (t0), follow-up (t2) versus after the intervention (t1), and follow-up (t2) versus baseline (t0). The arrows indicate the direction of change compared to the earlier time point in each comparison (up or down); the fill color represents the log_2_ paired fold change, indicating the size and direction of change (red to blue, with up or down changes shown by the colors). The asterisks indicate the significance level of the Benjamini–Hochberg-adjusted *P* value (*P < 0.05, **P < 0.01, ***P < 0.001, ****P < 0.0001). **b**,**d**,**f**, Combined box and line plots with the group trend line indicating concentrations of measured parameters at the three time points (switch to WD (**b**), switch to AHD (**d**) and fermented beverage (**f**)). Lines are grouped per participant. Cytokine production is presented as the log_2_ concentrations of IFNγ, IL-10, IL-1β, IL-6 and TNF in the supernatant. In all box plots, the box line defines the median level, the hinges depict the 25th and 75th centiles and the whiskers extend to ±1.5 times the IQR. ^a^Change in control group.

LMM analysis revealed that cytokine responses in participants switching from an AHD to a WD declined in 12 and 14 measures after the intervention and at the follow-up, compared to baseline, respectively, while four measures increased after the intervention compared to baseline. Despite elevated plasma inflammatory proteins (Fig. 2a), TNF and IFNγ responses to all stimuli, except poly(I:C), decreased after the intervention (Fig. 3a, Supplementary Fig. 4a and Supplementary Table 8), which is consistent with reduced cytokine production commonly observed in inflammatory conditions such as aging^17^, obesity^18^, endotoxemia^19^ or sepsis^20^. The largest decline was observed for *C. albicans* responses, especially in IFNγ, but also in other cytokines (Fig. 3b). IL-1β and IFNγ responses to poly(I:C) initially increased after the intervention, but this effect was not sustained at the follow-up (Fig. 3a and Supplementary Fig. 4a). IL-6 responses to LPS, *S. aureus*, *C. albicans*, MTB and *S. enteritidis* decreased at the follow-up compared to baseline (Fig. 3a).

Conversely, switching from a WD to an AHD resulted in only few changes (Fig. 3c, Supplementary Fig. 4b and Supplementary Table 8). At the follow-up, TNF, IL-1β, and IFNγ responses to *S. aureus* and TNF responses to *S. enteritidis* were decreased compared to both baseline and after the intervention. In contrast, IL-1β responses to MTB showed an increase at the follow-up compared to baseline and after the intervention (Fig. 3c,d). Finally, the fermented beverage group showed increased responses of the anti-inflammatory cytokine IL-10 after the intervention and at the follow-up compared to the baseline, alongside increased TNF, IL-1β, IL-6 and IL-10 responses to *C. albicans* (Fig. 3e,f, Supplementary Fig. 4c and Supplementary Table 8). In contrast, the transitioning to a WD led to a marked decline in *C. albicans* responses.

In summary, the interventions had notable effects on cytokine production. Switching to a WD reduced TNF and IFNγ production, while the fermented beverage increased anti-inflammatory IL-10 responses and enhanced *C. albicans* cytokine responses.

### Plasma metabolome

**Extended Data Fig. 4:**
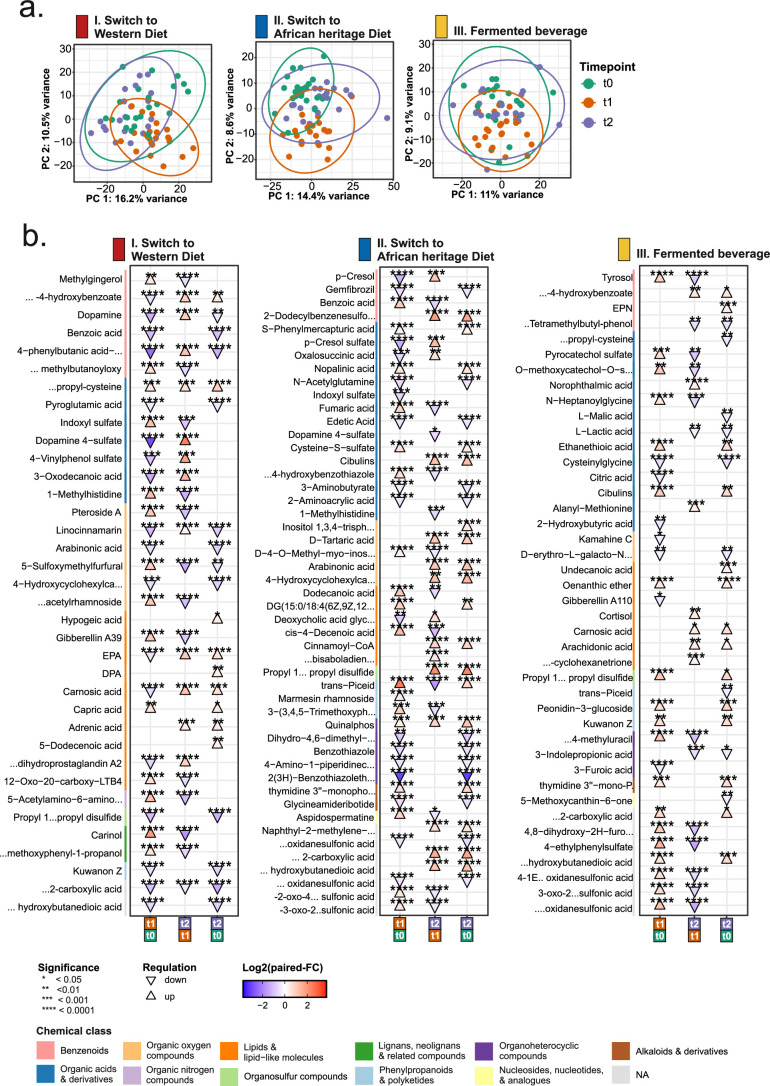
Differentially abundant plasma metabolites in the intervention groups. Plasma metabolome was performed using untargeted metabolomics (General Metabolics, Cambridge, MA) to assess changes during the transition to a Western diet (WD), African heritage-style diet (AHD) and fermented beverage consumption. Samples were obtained at baseline (t0) post-intervention (t1, week 2 for the WD or AHD diet interventions and one week for the fermented beverage intervention, N = 22/group), and after four weeks follow-up (t2, N = 22/group). **a**- Dotplots showing principal component 1 (PC1) and PC2 from principal components analysis (PCA) of log2-scaled ion intensity scaled values using all metabolites as input. Color indicates the time points (green, orange, purple for t0, t1 and t2, respectively). A geometric ellipse is drawn at 95% confidence for each time point. **b**- Triangle plots present top differentially abundant metabolites (DAMs, adjusted P value < 0.05 and absolute paired fold-change > 1.2). Arrows’ direction (up or down) indicates the direction of change, comparing late vs. early time points in each comparison as listed at the bottom of the plot, with color fill depicting the log2 paired fold-change value for each comparison. Statistical analysis was carried out using a linear mixed model for each comparison, including time point, BMI, physical activity level and age as fixed variables, and participant ID as random effect. Asterisks indicate significance level based on BH-adjusted P value (*, **, ***, **** represents adj. P values < 0.05, 0.01, 0.001, 0.0001).

Diet influences plasma metabolome composition^21^, with immunomodulatory effects from food-derived and microbiome-derived metabolites. We analyzed the plasma metabolome, a prespecified primary outcome, using untargeted metabolomics, identifying 1,266 ions and 1,339 molecular formulas, with 935 metabolites annotated in the Human Metabolome Database (HMDB) (Methods and Supplementary Table 9). PCA revealed altered metabolic profiles after the intervention across all arms compared to baseline (Extended Data Fig. 4a). An LMM analysis was applied to detect differentially abundant metabolites (DAMs) (absolute fold change greater than 1.2, *P*_adj_ < 0.05), focusing on metabolites where time points accounted for more than 5% of the variance (Supplementary Table 10). DAMs observed in control participants were reported in the downstream analysis (Supplementary Fig. 5 and Supplementary Table 10). Metabolites were classified into 14 chemical groups; pathway enrichment analysis was conducted using the Relational Database of Metabolomics Pathways (RaMP) (https://rampdb.nih.gov/), focusing on WikiPathways terms (1,377 pathways, 3,966 metabolites and 14,191 metabolite–pathway associations) to identify metabolites within or influencing specific pathways (Supplementary Table 11)^22^.

**Fig. 4:**
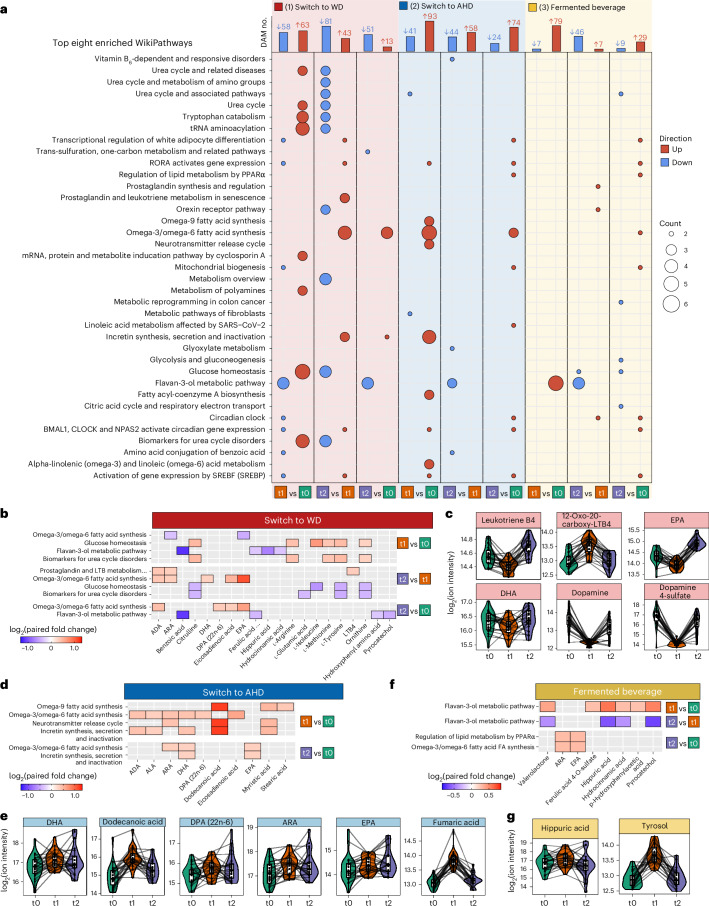
Pathway enrichment analysis for plasma DAMs. **a**, For each dietary arm and time comparison, the top bar plots present the number of DAMs at each time point. An LMM was used for each comparison with a two-sided hypothesis test. Fixed effects included time point, baseline BMI, age and baseline physical activity level; participant ID was included as a random effect. Significance level was set to Benjamini–Hochberg-adjusted *P* ≤ 0.05 and absolute paired fold change greater than 1.2. The lower dot plots present the top eight significantly enriched WikiPathway metabolic pathways for DAMs using a Fisher’s exact test via the RaMP database. The color indicates the direction of the DAM, up or down in red or blue, respectively. The size of the dots depicts the number of metabolites related to the pathway. Pathways are ordered alphabetically. **b**,**d**,**f**, Heatmaps showing examples of the enriched pathways discussed in the main text for switch to WD (**b**), switch to AHD (**d**) and fermented beverage (**f**), with the fill color indicating the relevant log_2_ paired fold change. **c**,**e**,**g**, Respective associated DAMs for switch to WD (**c**), switch to AHD (**e**) and fermented beverage (**g**) are shown in combined box and line plots with the group trend line indicating concentrations of measured parameters at the three time points. Lines are grouped per participant. In all box plots, the line defines the median value, the hinges depict the 25th and 75th centiles and the whiskers extend to ±1.5 times the IQR. ADA, adrenic acid; ARA, arachidonic acid; DHA, docosahexaenoic acid; DPA, docosapentaenoic acid; EPA, eicosapentaenoic acid; LTB4, leukotriene B4; ALA, alpha-linolenic acid; Valerolactone, 5-(hydroxyphenyl)-gamma-valerolactone-O-sulphate.

Transitioning from the AHD to WD resulted in 309 DAMs. Among these, 63 metabolites increased and 58 decreased after the intervention compared to the baseline, while 13 increased and 51 decreased at the follow-up compared to the baseline (Fig. 4a). Furthermore, 43 metabolites increased and 81 decreased at the follow-up compared to after the intervention. Top DAMs and their chemical classes are shown in Extended Data Fig. 4b. Pathway enrichment analysis revealed increases in metabolites linked to ‘glucose homeostasis’, the urea cycle (for example, ‘biomarkers for urea cycle disorders’), ‘tRNA aminoacylation’ and ‘tryptophan catabolism’ after the intervention (Fig. 4a and Supplementary Table 11), presumably reflecting the higher animal protein content in the WD, also indicated by increases in metabolites as 1-methylhistidine. Conversely, metabolites linked to the ‘flavan-3-ol’ pathway, a flavonoid subclass, decreased. The ‘omega-3/omega-6 fatty acid synthesis’ pathway was enriched at the follow-up compared to after the intervention and baseline. The omega-3 polyunsaturated fatty acid eicosapentaenoic acid (EPA) significantly decreased after the intervention compared to the baseline, while docosahexaenoic acid (DHA) showed a trend toward decreasing *(P* = 0.09), with both increasing again at the follow-up (Fig. 4b,c and Supplementary Table 11). Arachidonic acid (ARA) and its pro-inflammatory metabolite leukotriene B4 transiently decreased after the intervention and significantly increased at the follow-up, alongside a rise in 20-carboxy leukotriene B4 after the intervention (Fig. 4b,c). Lastly, dopamine and dopamine sulfate transiently decreased after the intervention, which is consistent with studies linking high-fat diets to hypodopaminergic effects^23^.

Switching from a WD to an AHD resulted in 334 DAMs. Among these, 93 metabolites increased and 41 decreased after the intervention, while 74 increased and 24 decreased after the intervention compared to the baseline (Fig. 4a). The top increased DAMs after the intervention compared to the baseline included the fatty acids dodecanoic acid (lauric acid) and *cis*-4-decenoic acid (Extended Data Fig. 4b). Dodecanoic acid was suggested to improve mitochondrial function and glucose and lipid metabolism^24^. Additional DAMs that increased included benzoic acid, trans-piceid (a resveratrol glycoside with cardiovascular benefits)^25^ and fumaric acid (a tricarboxylic acid cycle intermediate and immunomodulator)^26^. In the controls, with the exception of benzoic acid, these metabolites were not differentially abundant or were only observed as differentially abundant at the follow-up compared to the baseline (Supplementary Fig. 5 and Supplementary Table 10). Among the top DAMs that decreased after the intervention were cresol, p-cresol sulfate and indoxyl sulfate, which returned to baseline levels at the follow-up. The latter two metabolites, produced via tyrosine and tryptophan fermentation by gut bacteria, are known to decrease with vegetarian diets^27^. Pathway enrichment analysis linked increased DAMs, such as the omega-3 polyunsaturated fatty acids docosahexaenoic acid (DHA), docosapentaenoic acid (DPA) and EPA, as well as arachidonic acid, dodecanoid acid and fumaric acid, to ‘omega-9 fatty acid synthesis’, ‘omega-3/omega-6 fatty acid synthesis’, ‘neurotransmitter release cycle’ and ‘incretin synthesis, secretion and inactivation’ pathways, with varying roles depending on the metabolite (Fig. 4a,d,e and Supplementary Table 11).

Consumption of the fermented beverage resulted in 177 DAMs, with 79 metabolites increasing and seven decreasing after the intervention compared to the baseline, and 29 increasing and nine decreasing at the follow-up compared to the baseline (Fig. 4a and Supplementary Table 10). Notable increases were observed in metabolites of the ‘flavan-3-ol metabolic pathway’, including hippuric acid, hydrocinnamic acid, p-hydroxyphenylacetic acid, pyrocatechol and valerolactone-O-sulfate (Fig. 4f,g, Extended Data Fig. 4b and Supplementary Table 11). These metabolites, formed by gut microbial breakdown of dietary polyphenols, have been associated with vascular health and anti-inflammatory effects^28^. The sustained decrease in cysteinylglycine (a glutathione breakdown product) and transient increase in tyrosol (a microbial metabolite of polyphenols), and organic sulfates like 4-ethylphenyl sulfate, may highlight microbial fermentation effects (Fig. 4g and Extended Data Fig. 4b)^29^.

In summary, the dietary switch and fermented beverage both had distinct effects on metabolic pathways related to glucose, lipid, fatty acid and amino acid metabolism, and pathways associated with inflammation.

### Whole-blood transcriptome

**Extended Data Fig. 5:**
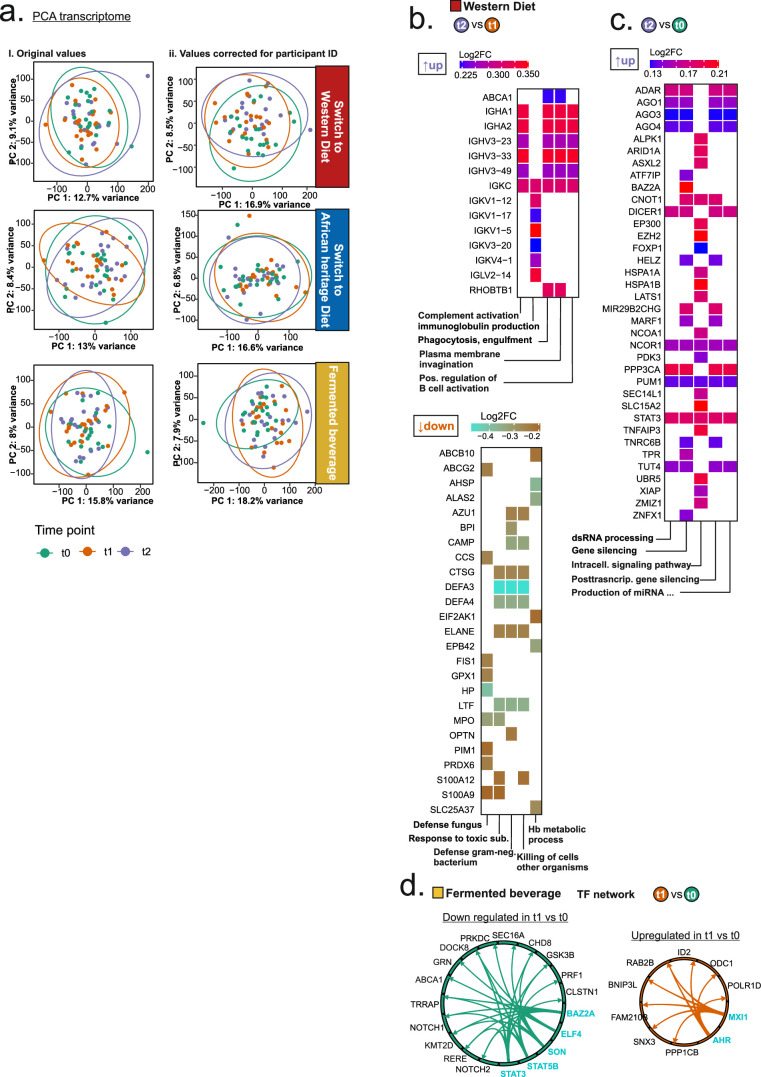
Whole blood transcriptome PCA and enrichment analysis. Whole blood transcriptome data were analysed for participants in the heritage to Western diet switch group (Switch to WD, N = 22), the Western to heritage diet switch group (Switch to AHD, N = 22) and the fermented beverage group (N = 21). Blood samples were collected at 3 time points: baseline (t0), post-intervention (t1), and follow-up (t2), four weeks after the end of intervention. Five control individuals for each dietary arm were included in the DE analysis using DESeq2, with model design including the individual (ind.n), treatment (diet or control) and the time point (baseline, post-intervention or follow-up): ~ treatment + treatment:ind.n + treatment:time point. DE analysis was performed between each two time points using the Benjamini-Hochberg (BH) controlling method for *P* value adjustment and independent hypothesis weighting (IHW). The two-sided significance level was set to adjusted *P*_adj_ ≤ 0.2, with no fold change threshold, **a**, PCA was calculated on scaled variance stabilized transformed (vst) count values. Dot plots depict the data on based on principal component (PC)1 and PC2 (x and y-axis respectively). Dot colors represent time points: t0 (green), t1 (orange), and t2 (purple). **b, c** - Tile plots display the log2 fold-change (log2FC) of genes related to selected GO terms enriched in the switch to the Western Diet (WD) group (indicated by color). **d**- Circular plot shows the predicted transcription factors (TF) and their targets among upregulated genes in the fermented beverage intervention group (t1 vs t0, based on ChEA3 analysis). Gene names are displayed around the circle’s perimeter, with turquoise marking the TF, and arrows indicating the predicted regulated genes.

We also analyzed changes in the prespecified primary outcome of whole-blood transcriptome. We performed differential expression analysis (*P*_adj_ < 0.2, no fold change cutoff), considering both the individual and control group of each intervention arm (Methods). PCA of the transcriptome alterations revealed subtle shifts across the first (PC1) and second (PC2) PCs in the participants switching to a WD and those in the fermented beverage group intervention, both after the intervention and at the follow-up, compared to the baseline (values corrected for participant; Extended Data Fig. 5a).

**Fig. 5:**
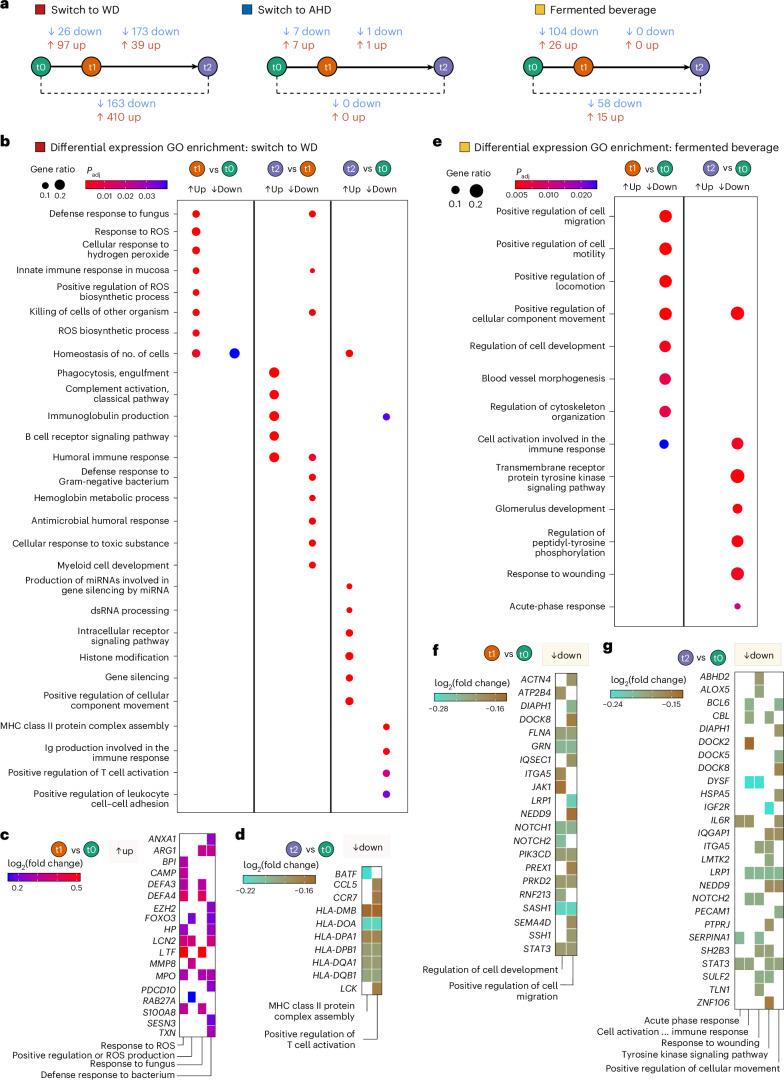
Whole-blood transcriptome differential expression analysis. Whole-blood transcriptome data were analyzed for participants in the AHD to WD switch group (*n* = 22), the WD to AHD switch group (*n* = 22) and the fermented beverage group (*n* = 21). Blood samples were collected at three time points: baseline (t0), after the intervention (t1) and the follow-up (t2), 4 weeks after the end of the intervention. Five control individuals for each dietary arm were included in the differential expression analysis, using DESeq2. The model design incorporated the individual (ind.*n*), intervention (diet versus control) and time point (baseline, after the intervention or at the follow-up) as follows: ~intervention + intervention:ind. *n* + intervention:time point. Differential expression analysis was performed between time points using the Benjamini–Hochberg method for *P* value adjustment and independent hypothesis weighting (IHW). A two-sided significance threshold was set at adjusted *P* value ≤ 0.2, with no fold change cutoff. **a**, A scheme presenting the number of DEGs for each comparison in each of the intervention arms are presented. **b**,**e**, Dot plots presenting the results of gene set overrepresentation analyses for GO terms performed with the DEGs from each comparison for the switch to WD (**b**) and fermented beverage (**e**). The size and color of the dots depict the gene ratio and Benjamini–Hochberg-adjusted *P*, respectively. **c**,**d**,**f**,**g**, Tile plots presenting the log_2_ fold change of the genes related to selected enriched GO terms for switch to WD (after intervention (t1) versus baseline (t0) (**c**) and follow-up (t2) versus baseline (t0) (**d**)), and the fermented beverage (after intervention (t1) versus baseline (t0) (**f**) and follow-up (t2) versus baseline (t0) (**g**)).

The switch from the AHD to WD resulted in the most differentially expressed genes (DEGs), with 97 upregulated and 26 downregulated after the intervention compared to the baseline, and 410 upregulated and 163 downregulated at the follow-up compared to the baseline (Fig. 5a). In contrast, the switch from WD to AHD had minimal effects, with seven upregulated and seven downregulated genes after the intervention compared to the baseline, and no differences at the follow-up compared to the baseline. Fermented beverage consumption resulted in 26 upregulated and 104 downregulated DEGs after the intervention compared to the baseline and 15 upregulated and 58 downregulated genes at the follow-up compared to the baseline (Fig. 5a and Supplementary Tables 12–14).

In participants switching to a WD, upregulated genes after the intervention were enriched for Gene Ontology (GO) terms related to reactive oxygen species (ROS), including ‘response to ROS’, ‘cellular response to hydrogen peroxide’ and ‘positive regulation of ROS production’, and pathogen defense terms such as ‘response to fungus’ and ‘defense response to bacterium’ (Fig. 5b,c and Supplementary Table 15). Key genes included *S100A8*, *MPO*, *MMP8* and *DEFA3* and *DEFA4*, all strongly expressed in neutrophils^30^. In contrast, downregulated DEGs at the follow-up compared to the baseline were related to adaptive immunity, showing enrichment in processes such as ‘immunoglobulin production’, ‘positive regulation of T cell activation’ and ‘MHC class II protein complex assembly’, including several human leukocyte antigen (HLA) genes (Fig. 5b,d and Supplementary Table 15). Upregulated genes at the follow-up compared to the baseline were associated with posttranscriptional modification terms, such as ‘production of miRNA involved in gene silencing’ and ‘histone modification’ (Fig. 5b and Extended Data Fig. 5c).

In the fermented beverage group, downregulated genes after the intervention compared to the baseline were linked to cellular migration processes, including ‘positive regulation of cell migration’ and ‘regulation of cell development’, including genes such as *ACTN4*, *STAT3*, *DIAPH1*, *NOTCH1* and *NOTCH2*, *LRP1*, *JAK1* and *SEMA4D* (Fig. 5e,f and Supplementary Table 16). Transcription factor enrichment analysis identified STAT3, STAT5B and SON DNA binding protein (an intracellular infection mediator in macrophages)^31^, and ELF4 (a known regulator of IFN induction)^32^, as key regulators of downregulated DEGs (Extended Data Fig. 5d and Supplementary Table 17). Moreover, aryl hydrocarbon receptor expression encodes a transcription factor responsive to metabolites such as indole-lactic acid and from fermented foods; its targets were increased (Extended Data Fig. 5d and Supplementary Table 17)^33^. Comparing the follow-up with the baseline, a decrease in genes related to inflammation progression and resolution pathways was observed. This included pathways such as ‘cell activation involved in immune response’, ‘response to wounding’, ‘acute phase response’ and ‘positive regulation of cellular component movement’, with key genes such as *IL6R*, *DOCK2*, *DOCK5*, *DOCK8*, *DIAPH1* and *LRP1* (Fig. 5e,g and Supplementary Table 16).

In summary, these results support that a WD is associated with upregulation in genes of the innate immune system, whereas the fermented banana beverage induced a more anti-inflammatory transcriptional profile.

### Safety and adverse events

Possible adverse events were monitored through spontaneous reporting because the risks of the short-term dietary interventions were considered minimal. Participants were instructed to report any adverse events to the study team, but none occurred.

### Post-hoc analyses of circulating leukocyte activation

**Fig. 6:**
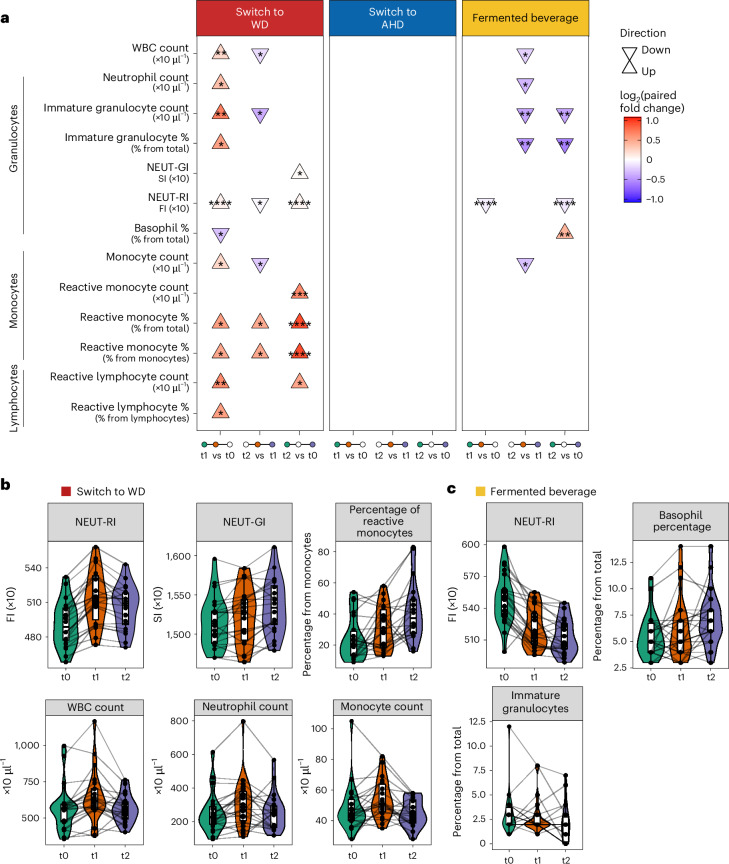
Changes in the number and activation status of circulating white blood cells. **a**, Triangle plot presenting the significantly different leukocyte parameters across the baseline (t0), after the intervention (t1, 2 weeks for diets and 1 week for fermented beverage) and at the follow-up (t2, 4 weeks after t1) time points (*n* = 22 from each diet). Parameters were measured using the XN-450 hematology analyzer (Sysmex). The direction of the arrows (up or down) indicates the direction of change, comparing late versus early time points in each comparison as listed at the bottom of the plot. An LMM was used for each comparison, with a two-sided hypothesis test. Fixed effects included time point, baseline BMI, age and baseline physical activity level, while participant ID was included as a random effect. The asterisks indicate the significance level based on Benjamini–Hochberg-adjusted *P* values (*P < 0.05, **P < 0.01, ***P < 0.001, ****P < 0.0001). **b**,**c**, Combined violin, box and line plots depicting the values of the measured parameters (switch to WD (**b**) and fermented beverage (**c**)) across the three time points with participant-specific lines linking observations. In all box plots, the central line represents the median, the hinges indicate the 25th and 75th centiles, and the whiskers extend to ±1.5 times the IQR. NEUT-RI and NEUT-GI values are expressed in fluorescence intensity (FI) units and scatter intensity (SI) units, respectively. WBC, white blood cell count.

We conducted a post-hoc analysis to assess the intervention’s impact on circulating leukocyte subsets and activation status. Using a Sysmex XN-450 hematology analyzer with its extended inflammation parameters, we measured complete blood counts and activation parameters for lymphocytes (reactive lymphocytes (RE-LYMPHs)), monocytes (reactive monocytes (RE-MONOs)) and neutrophils (neutrophil reactivity intensity (NEUT-RI) and neutrophil granularity intensity (NEUT-GI))^34^. LMM analysis revealed significant leukocyte changes, predominantly in participants switching to a WD, with smaller effects in participants consuming the fermented beverage (Fig. 6a and Supplementary Table 18). Switching to a WD increased white blood cells, neutrophils, immature granulocytes, NEUT-RI, monocytes, RE-MONOs and RE-LYMPHs from baseline to after the intervention (Fig. 6a,b). While white blood cell counts returned to baseline levels at the follow-up, activation markers (NEUT-RI, NEUT-GI, RE-MONOs, RE-LYMPHs) remained elevated, suggesting persistent immune cell activation. Conversely, switching to an AHD showed no significant leukocyte changes. The fermented beverage reduced NEUT-RI after the intervention, with effects persisting at the follow-up, alongside lower immature granulocytes and higher basophil percentages (Fig. 6a,c). No significant changes were observed in the control participants maintained on their habitual diet (Supplementary Table 18).

Overall, the intervention substantially influenced leukocyte numbers and phenotype, with the WD promoting myelopoiesis and immune cell activation, while the fermented beverage reduced neutrophil activation.

### Independent validation via cross-sectional cohort

**Extended Data Fig. 6:**
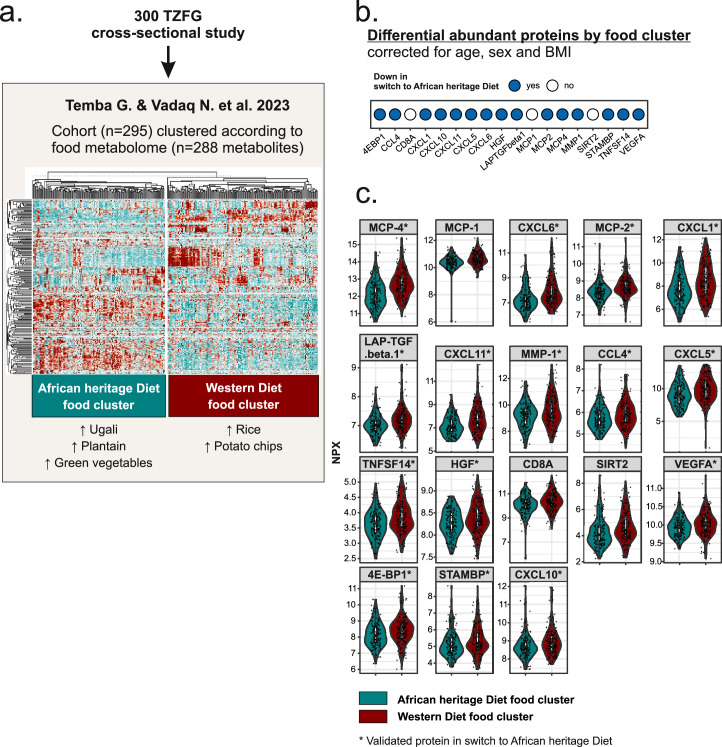
Plasma proteome differences in the cross-sectional 300 Tanzania Functional Genomics (300 TZFG) project align with changes observed in the heritage diet switch group. Results from the 300 TZFG cross-sectional cohort were used to validate the findings in the dietary intervention study. **a**- Participants in the 300 TZFG cohort (*n* = 295) were assigned, based on their food-derived metabolome profile, to a food cluster associated with a more African heritage-style diet (AHD food cluster; *n* = 138) or a more Western diet (WD food cluster; *n* = 157), as previously described^9^. **b**- Results of the Olink 92 inflammation panel were compared between the African heritage diet and Western diet food clusters using a linear model, correcting for age, sex and BMI. The dot plot shows differentially expressed proteins between the two clusters, with blue indicating proteins that also exhibited significant changes in the Western to heritage diet intervention arm of the current study. **c**- Combined violin-box-scatter plots present the levels of measured parameters across the two food clusters in the 300 TZFG cohort. Proteins levels are presented as NPX values.

To validate the findings, we analyzed data from the 300 Tanzania functional genomics (TZFG) cohort, a cross-sectional study of urban- or rural-living Tanzanians (*n* = 295) from the same region. Participants were previously categorized into ‘Kilimanjaro heritage-style diet’ (*n* = 138) and ‘Western-style diet’ clusters (*n* = 157) based on food-derived plasma metabolome profiles (Extended Data Fig. 6a)^9^. Differential analysis using the Olink inflammation panel identified 18 proteins that were lower in the heritage-style diet cluster compared to the Western-style diet cluster (Supplementary Table 19). Among these, 83% (15 proteins) also decreased in the current WD to AHD arm (Extended Data Fig. 6b,c and Supplementary Table 19). These proteins included the chemokines CXCL1, CXCL5, CXCL6, CXCL11, MCP2 and MCP4.

## Discussion

AHDs are rapidly being replaced by WDs across the continent. In a randomized controlled trial, we found that switching to a WD impacted key metabolic pathways linked to NCDs and induced a pro-inflammatory state involving leukocytes, inflammatory mediators and gene expression. In contrast, the switch to a largely plant-based heritage diet typical for the Kilimanjaro region and the fermented beverage primarily induced anti-inflammatory effects. Some of these changes persisted for 4 weeks after the intervention, demonstrating a sustained impact. This study highlights the immunometabolic consequences of dietary transitions and suggests their potential effects beyond a short dietary intervention.

The heritage diet in this study is rich in green vegetables, legumes, plantains, root and tuber crops, including cassava and taro, and whole grains (millet, sorghum), providing dietary fiber and bioactive compounds like polyphenols, known for their anti-inflammatory properties and beneficial metabolic effects^3,35–39^. Additionally, this diet promotes a favorable gut microbiome composition and production of beneficial metabolites^40^. Our plasma proteomics and metabolomics analyses revealed changes in several classes of immunomodulating compounds and metabolic pathways, including microbiota-derived metabolites. Notably, in the AHD group, inflammatory proteins decreased without changes in circulating leukocytes or gene transcription, suggesting that its anti-inflammatory effects stem from modulation of metabolic processes in tissues and possibly the microbiome, rather than leukocyte transcriptional regulation. The immunometabolic benefits of this diet probably stem from synergistic interactions among diverse foods and bioactive compounds, rather than any single component, a pattern also seen with the Mediterranean diet^41^.

The WD, rich in calorie-dense processed foods, saturated fats, salt and refined carbohydrates, promotes inflammation. This probably results from both direct immunometabolic effects of cholesterol, saturated lipids and sugars, and indirect effects via gut dysbiosis and weight gain^6,42^.

Our study also highlights the potential health benefits of traditional African fermented beverages^43^. The Chagga tribe’s heritage beer, Mbege, is prepared by fermenting boiled bananas with germinated finger millet porridge (*Eleusine coracana*). The beverage has an alcohol content of 1–3%, depending on the duration of fermentation^12,44^. Besides the health-promoting compounds naturally present in bananas and millet, fermentation enriches Mbege with bioactive metabolites and microbes, such as *Saccharomyces cerevisiae* and lactobacilli^44^. Consumption of Mbege reduced, among others, neutrophil activation and boosted cytokine responses to *Candida*. We previously showed that β-glucan from *S. cerevisiae* is a potent inducer of trained immunity^45^. Metabolites like tyrosol, produced by *S. cerevisiae*^46^, were elevated in the participants in the Mbege arm, and therefore support its immunomodulating effects. Moreover, the flavan-3-ol pathway, which involves a subclass of polyphenols with recognized vascular benefits^28^, emerged as one of the most upregulated metabolic pathways in the Mbege arm. However, consumption of these fermented beverages should be balanced against risks such as the alcohol content and potential mycotoxin contamination.

Chronic inflammation is a key driver of many NCDs^8^; studies have linked low-inflammatory diets to reduced risks of CVD, type 2 diabetes and cancer^47,48^. Adherence to the Mediterranean diet has shown benefit in both primary and secondary prevention of CVD^49,50^. Our study found that even brief dietary interventions can have prolonged effects on the immune and metabolic systems. While we cannot entirely rule out that some sustained effects were due to continued adherence to the intervention diets, participants were instructed to revert to their habitual diets after the intervention. Additionally, the limited availability of Western-style food products in rural areas makes continuous consumption of a WD unlikely. The persistent changes in neutrophil count and activation, inflammatory and cardiometabolic proteins, and transcription of immune-related genes at 4 weeks after the intervention align with prior experimental murine studies showing that the WD induces a maladaptive ‘trained immunity’ program and that alternating high-fat diets stimulate myelopoiesis, increasing neutrophil counts and progenitor reprogramming, thereby promoting atherosclerosis^7,51^, The role of neutrophils in CVD is increasingly recognized^52^. Notably, the Mediterranean diet has been reported to reduce neutrophil counts and carotid intima-media thickness^53^.

The rapid rise in obesity and NCDs across Africa underscores the need for sustainable, region-specific interventions. Preserving the consumption of heritage diets or incorporating indigenous and traditional foods into dietary practices could have a critical role in preventing and managing NCDs. However, Africa’s diverse heritage diets are largely understudied, and the field of nutritional immunology in this context is still emerging. Policymakers often rely on findings from the Global North, which do not account for regional dietary, cultural and genetic differences. Our study provides direct evidence of the health risks of dietary westernization and the benefits of heritage diets and indigenous beverages, emphasizing the need for further research to explore their health impacts before they are lost to globalization, ensuring culturally appropriate and sustainable public health solutions.

Our study also has a few limitations. First, food was offered ad libitum, and the intervention was not isocaloric, resulting in a weight gain in the AHD to WD switch group, which may have influenced immunometabolic profiles^54^. Second, the dietary intervention was short, although similar or even shorter interventions have demonstrated important immune effects. For example, a 2-week intervention with a vegan or ketogenic diet induced marked immune and gut microbiome changes^4^, while a 5-day fiber-poor diet was sufficient to alter host responses in both mice and humans^55^. Furthermore, our controlled setting established a baseline for short-term dietary responses in African environments, laying the foundation for future research on long-term effects. Third, our study included only men because of cultural logistics and confounding variables. Future studies should include women and diverse age groups. Fourth, the sample size is relatively small, although it is comparable to other multiomics dietary studies^4,5^. Sample size calculations were based on functional immune responses rather than multiomics data, given the challenges in defining meaningful effect sizes for large, complex multiomics datasets. The homogeneous study population and the largely consistent direction of inflammatory and metabolic changes in the intervention groups, reduce the likelihood that our findings are due to chance. Fifth, the study only provided molecular insights into short-term changes in immunometabolic features and pathways because of dietary practices but did not assess long-term clinical outcomes. Sixth, plasma metabolomics analysis used a nontargeted, high-throughput method relying on *m/z* ratios, which may lack specificity for definitive metabolite identification. Lastly, while the Chagga tribe’s plant-rich and fiber-rich diet aligns with healthy dietary practices, Tanzania has over 100 tribes with varied diets. Thus, findings may not be generalizable to all heritage diets in the region.

In summary, our findings highlight the substantial impact of dietary changes on both immune and metabolic systems, with implications for infection susceptibility and NCDs. They underscore the importance of preserving essential elements of AHDs, including fermented products, to mitigate the rising burden of NCDs and immune dysregulation in sub-Saharan Africa. As WDs increasingly replace indigenous diets, documenting the health benefits of heritage diets remains important.

## Methods

### Study area

This study was conducted in the Moshi district in the Kilimanjaro region of northeastern Tanzania between April and August 2021, which coincides largely with the wet season. Moshi town serves as the administrative, commercial and educational center for the region, with a population of approximately 220,000 inhabitants. Moshi town borders the foothills of the southern slopes of Mount Kilimanjaro. These foothills are rural areas where people live in smaller villages and are engaged in subsistence agriculture and animal husbandry. Most of the people living in the foothills belong to the Chagga tribe. For this study, rural dwellers were mainly recruited from the village of Uru Shimbwe Juu, which is located approximately 15 km from Moshi town at an elevation of 1,978 m.

### Participants and eligibility

Two cohorts of healthy volunteers assigned male at birth were recruited: (1) males residing in the rural village of Uru Shimbwe Juu or its environs, adhering to a predominantly Kilimanjaro heritage diet; and (2) males residing in Moshi town, adhering to a predominantly Western-style diet. Sex was determined based on self-report. Information about the study was disseminated through leaflets, community talks and announcements during church gatherings. Dietary habits in eligible volunteers who agreed to participate were assessed using a dietary recall questionnaire, conducted over three nonconsecutive 24-h periods in 1 week, including 1 weekend day. The questionnaire captured details on different foods, as well as estimated portion sizes (small, medium or large) and preparation methods for each food item. Questionnaires were evaluated by a nutritionist to ensure participants were adhering to either a diet typical of the Kilimanjaro region or a Western-style diet. This assessment relied primarily on the nutritionist’s expertise and knowledge of local dietary habits, rather than a standardized set of criteria, incorporating traditional understanding of food patterns.

Only participants adhering to either a Kilimanjaro heritage-style diet or Western-style diet were further considered for participation. Other inclusion criteria were age 20–40 years, BMI between 18 and 25 kg m^−^^2^, residing in either the rural or urban areas of Moshi for a minimum of 1 month preceding the study, and commitment to stay in the study area throughout the intervention period. Exclusion criteria were a positive result of human immunodeficiency virus or malaria rapid test, notable blood pressure abnormalities, elevated fasting blood sugar level, food allergies, recent acute illness, use of medication, antibiotics or vaccination in the last 3 months, previous hospitalization within the past year, presence of chronic conditions such as active malignancy, liver or kidney disease, tuberculosis, chronic hepatitis B or C infection, or unwillingness to consume alcohol. A standardized questionnaire was used to collect meta-data, lifestyle information, health status and daily activities, including tobacco use, physical activity level, medical history, medication use and vaccination records.

Participants’ physical activity was assessed by inquiring about their engagement in daily walking and sports activities. For descriptive purposes (Extended Data Table 1), sports activity levels were classified as follows: very active (more than 2 h of daily activity); active (up to 1 h daily or up to 2 h on some days); less active (up to 1 h on some days or only on weekends); and not active (no reported activity). Daily walking was categorized into three groups: less than 30 min; 30–60 min; or more than 60 min per day. For downstream analysis, we summarized these two estimations of physical activity with the scoring system provided in Supplementary Table 1. Briefly, engaging in any sports activity was scored as 2, and no sports activity as 1. The frequency of sports activity was scored as 1, 2 or 3 for ‘only weekend’, ‘some days in a week’ and ‘every day’, respectively. Duration of daily walking was scored between 1, 2 and 3 for less than 30 min, between 30 and 60 min and more than 60 min per day, respectively. Means of transportation was scored as 1 for private or public vehicle transportation or 2 for physical transportation (walking or bicycle). Scores values were scaled, and a sum of physical activity level was generated for each individual (Supplementary Table 5).

The current study was approved by the Ethical Committees of the Kilimanjaro Christian Medical University College (KCMUCo) (no. 2483) and the National Institute for Medical Research in Tanzania (no. NIMR/HQ/R.8a/Vol.IX 3570). Participant recruitment and experimental procedures were conducted according to the principles mentioned in the Declaration of Helsinki (2013). All participants gave written informed consent before recruitment.

### Design and procedures

The study was designed as an open-label, prospective intervention study with three arms: (1) rural-living men with a Kilimanjaro heritage diet who shifted to a Western-style diet for two weeks; (2) urban-living men with a Western-style diet shifting to a heritage diet for 2 weeks; and (3) men with a Western-style diet starting daily consumption of the fermented drink ‘Mbege’ for 1 week (fermented beverage arm). To assess for dietary-independent effects, five controls who remained on their habitual diet were also included in the first two arms.

### Randomization

Participants in the dietary intervention arms were randomized to either the dietary intervention (*n* = 23 in the AHD to WD arm; *n* = 22 in the WD to AHD arm) or remaining on their habitual diet (*n* = 5 in each arm, ‘controls’). No controls were included in the fermented beverage arm. Randomization was conducted by an independent statistician using a lottery method. Each preselected participant was assigned a number based on the dietary assessment, from which five numbers (controls) were randomly selected.

### Intervention diets and procedures

The switch group received freshly prepared meals from a cook three times daily (breakfast, lunch and dinner) at a central cooking location in either Uru Shimbwe Juu village or in Moshi town. Food was provided ad libitum and consumed under supervision. Portion sizes were not quantified. In the fermented beverage arm, participants were directed to consume at least 1 l of the beverage daily between 16:00 to 17:00 while maintaining their usual diet. The beverage, produced fresh daily by one local manufacturer, was dispensed at a central location in Moshi town and intake was supervised. Intake of this fermented beverage was restricted in the other groups. Blood samples were collected at baseline, after the intervention (2 weeks for the dietary switch, 1 week for the fermented banana beverage), and 4 weeks thereafter for the follow-up. For the rural group, samples were collected at the field site in Uru Shimbwe Juu, while for the urban group, samples were collected at the Kilimanjaro Clinical Research Institute (KCRI) research unit in Moshi town.

The dietary intervention diets are detailed in Extended Data Table 2. Using the Tanzania Food Composition Table^56^, we retrospectively calculated the nutrient composition of the intervention diets offered to participants. Because of the absence of recorded portion sizes, macronutrient contributions to energy intake were calculated per 100 g of food and adjusted based on typical daily intake, assuming standard portion sizes. In the WD, carbohydrates contributed approximately 55% of total energy, primarily from refined sources such as refined maize flour (used for making stiff porridge, ‘ugali’), pancakes, potato chips, white rice, macaroni and fried plantains. Fats accounted for 23% of total energy, mainly from fried foods like fried chicken, beef, potato chips, ‘chips mayai’ (a local dish combining potato chips and eggs) and pancakes. Protein contributed 16% of total energy, with chicken, beef and eggs as the primary sources. Simple sugars and dietary fiber contributed 2.2% and 3.5% of total energy, respectively. In contrast, the Kilimanjaro AHD provided 60% of total energy from carbohydrates, mainly derived from whole grains, roots and tubers, such as taro and boiled sweet potatoes. Protein contributed 11% of total energy, primarily from meat, local free-range chicken and fish. Fats accounted for 7% of total energy, while simple sugars and dietary fiber contributed 7% and 14% of total energy, respectively.

### Outcomes

The prespecified primary outcomes were the changes in (1) circulating inflammation-related biomarkers; (2) the capacity of the circulating immune cells to produce inflammatory cytokines in ex vivo whole-blood stimulation to different stimuli; (3) whole-blood transcriptome; and (4) untargeted plasma metabolome, at baseline, after the intervention and 4 weeks after the intervention. A secondary outcome was the change in gut microbiome composition, assessed through untargeted metagenomics. These results will be reported separately.

### Sample size calculation

The sample size for this study was determined based on functional immune response outcomes rather than high-dimensional omics data, which typically generate large datasets unsuitable for conventional power calculations. Specifically, the calculation was based on previously reported differences in whole-blood TNF production after LPS stimulation between urban and rural populations in a cross-sectional study conducted in the same region^11^. The sample size was calculated to achieve 80% statistical power to detect significant differences at a two-sided alpha level of 0.05.

### Safety

Regarding harm, safety and possible adverse events, the risk associated with participation in this short dietary intervention was considered minimal. Therefore, spontaneous adverse event reporting was applied. Participants were instructed to report any adverse events they experienced during the study period directly to the study team. In addition, body weight was monitored throughout the study.

### Protocol deviations

Fasting glucose and blood pressure data from the after the intervention and follow-up periods, although initially planned according to the study protocol, were not recorded because of logistical challenges. Difficult access to the remote study site on Mount Kilimanjaro during the rainy season led to delays that compromised the ability to obtain early-morning, fasting measurements.

### Hemocytometry

EDTA-anticoagulated blood samples were analyzed using the Sysmex XN analyzer (Sysmex Corporation) to obtain a complete blood count and the Sysmex Extended Inflammation Parameters, including RE-MONO, RE-LYMPH and NEUT-RI. Blood samples were analyzed at the KCRI in Moshi, Tanzania within 2 h after venipuncture.

Statistical comparisons across the study time points were performed using an LMM. The dream function (variancePartition package v.1.24) was applied to log_2_-transformed concentration values, with the time point as a fixed variable, participant as a random effect and adjustments for age, BMI and baseline physical activity level. Statistical summary results were extracted using the eBayes and topTable functions, applying false discovery rate (FDR) adjustment for multiple testing using Benjamini–Hochberg correction, with the significance cutoff for *P*_adj_ set to 0.05. Paired fold change values were calculated using the fcrosFCmat function from the package fcros (v.1.6.1). Statistical results for each dietary arm were presented in a triangle plot, with the direction of change and paired log_2_ fold change presented for each time point comparison using ggplot2 (v.3.5.1).

### Multiomics variance analysis

To determine the percentage of explained variance by the study intervention time point after the intervention (t1) or at the follow-up (t2) versus the baseline (t0) to the features in each data layer, we performed a variance partition analysis. Variance partition analysis was conducted using the fitExtractVarPartModel function from the variance partition package (v.1.24.1) using time point, age, BMI and physical activity level at baseline as fixed variables and participants’ ID as a random effect. Results were visualized in histograms using the ggplot2 and ggridges packages (v.3.5.1 and v.0.5.6, respectively).

### Whole-blood stimulation assays and cytokine quantification

The ex vivo cytokine stimulation experiments were conducted at the KCRI in Moshi, Tanzania. Whole-blood samples, obtained with heparin, were stimulated with bacterial, fungal and TLR3 and TLR4 agonists in a 48-well culture plate as described previously^11^. The following concentration stimuli were used: *E. coli* LPS (100 ng ml^−1^, Sigma-Aldrich); MTB (5 μg ml^−1^, H37Rv strain, in-house); poly(I:C) (50 µg ml^−1^, InvivoGen); *S. aureus* (10^6^ ml^−1^ colony-forming units (CFU), ATCC strain 29213, in-house); *C. albicans* (10^6^ CFU ml^−1^, UC820 strain, in-house); and *S. enteritidis* (10^6^ CFU ml^−1^, in-house). Stimuli were prepared in Roswell Park Memorial Institute culture medium (Dutch-modified, Invitrogen) supplemented with 50 µg ml^−1^ gentamicin, 2 mM GlutaMAX and 1 mM pyruvate. The resulting supernatants were stored at −80 °C. Cytokine concentrations in the supernatant were quantified using ELISA according to the manufacturer’s instructions provided by the respective kits (IL-6, IL-1β, IL-10 and TNF, R&D Systems; IFNγ, Sanquin). To ensure consistency, samples were measured using kits from the same lot. For statistical analysis, the cytokine levels measured in the whole-blood assay were log_2_-transformed.

Statistical comparisons across the study time points were performed using an LMM. The dream function (variancePartition package v.1.24) was applied to log_2_-transformed concentration values, with time point as a fixed variable, participant as a random effect and adjustments for age, BMI and baseline physical activity level. Statistical summary results were extracted using the eBayes and topTable functions, applying FDR adjustment for multiple testing using Benjamini–Hochberg correction, with significance cutoff for *P*_adj_ set to 0.05. Paired fold change values were calculated using the fcrosFCmat function from the package fcros (v.1.6.1). Statistical results for each dietary arm were presented in a triangle plot, presenting the direction of change and paired log_2_ fold change for each time point comparison using ggplot2 (v.3.5.1).

### Inflammatory and cardiometabolic plasma proteome

Plasma proteins were assessed using proximity extension technology (Olink Proteomics AB)^15^. The Olink 92 Inflammation and 92 Cardiometabolic panels were processed at the Laboratory of Experimental Internal Medicine, Radboudumc, the Netherlands. The assay involves the binding of target proteins to paired oligonucleotide antibody probes, followed by subsequent hybridization and amplification. NPX values, represented as arbitrary units on a log_2_ scale, were calculated from normalized cycle threshold data. Comprehensive validation details for the assay can be accessed on the Olink website (https://www.olink.com). All samples were measured in a single run in the same batch. Proteins with values below the limit of detection in more than 25% of subsequent samples were excluded. This resulted in the removal of 22 proteins, leaving 162 out of 184 proteins for further analysis. Most of these proteins had values below the limit of detection in almost all samples. Quality control measures and outlier detection were performed, revealing no significant batch effects or outliers attributable to technical issues. Additionally, control participants from both urban and rural groups exhibited consistent overall proteomic profiles, suggesting minimal batch-related bias. PCA was conducted using the prcomp function (stats v.3.6.2) on scaled NPX values or scaled NPX values corrected for participants (removeBatchEffect function, limma package v.3.50.3). To decrease the load of multiple testing, the proteins further tested in the differential analysis showed more than 5% of variance explained by the study time point (considering time point, age, BMI, physical activity level and participant as variables in the equation). The differential analysis for upregulated and downregulated proteins among the study time points was conducted using an LMM (variancePartition package v.1.24.) including time point as the main fixed variable, age, BMI and physical activity level at baseline as covariates, and the individual as a random effect (dream function). Statistical summary results were extracted for each time point comparison using the eBayes and topTable functions, FDR-adjusted for multiple testing using the Benjamini–Hochberg correction, setting the significance cutoff for *P*_adj_ ≤ 0.05. Paired fold change values were calculated using the fcrosFCmat function from the package fcros (v.1.6.1), calculating the fold changes as a trimmed mean of the fold changes obtained using pairs of samples. The statistical results for each dietary arm were summarized in a triangle plot, presenting the direction of change and paired log_2_ fold change for each time point comparison using ggplot2 (v.3.5.1). In addition, beyond protein assignment to the designated Olink panels, DAPs were annotated to biological processes, based on biological process information obtained from the Human Protein Atlas (v.23.0).

Plasma concentrations of highly sensitive CRP were measured using an ELISA, according to the manufacturer’s instructions (Quantikine ELISA Kit, R&D Systems). For samples with CRP levels below the detection limit, a value equivalent to the lower limit of detection (0.2 mg l^−1^) was assigned. A comparative analysis of CRP among the study time points in each study arm was conducted using an LMM (variancePartition package v.1.24.), with statistical summary results extracted for each time point comparison using the eBayes and topTable functions, FDR-adjusted for multiple testing using Benjamini–Hochberg correction, setting the significance cutoff for *P*_adj_ at 0.05.

### Proteomic profile cross-validation with the 300 TZFG cohort

Results from the 300 TZFG cross-sectional cohort were used to validate the findings in the dietary intervention study. This validation was based on previously published plasma metabolome and plasma proteome data^9^. Individuals from the 300 TZFG cohort (*n* = 295) were previously classified into two food clusters based on their food-associated plasma metabolome profiles: a Kilimanjaro heritage diet food cluster (*n* = 138) and a Western-type diet food cluster (*n* = 157). Differential proteins were detected using a linear model correcting for age, sex and BMI with the variancePartition package dream function followed by eBayes and topTable functions to extract summary statics (v.1.24). Differential proteins were visualized in combined violin-box-scatter plots, presenting the NPX values measured for each food cluster using ggplot2 (v.3.5.1). A qualitative comparison was conducted between the differential proteins in the 300 TZFG cohort and those in the heritage diet dietary arm, with results presented in a colored dot plot indicating proteins associated with the heritage diet in both datasets.

### Untargeted plasma metabolomics

Untargeted plasma metabolomics was performed by General Metabolics as described previously^57^. Briefly, an untargeted metabolomics approach was used to analyze plasma metabolites via high-throughput mass spectrometry (Agilent Technologies). The high-throughput method used is based on flow injection, without separation of compounds using a liquid chromatography gradient, allowing for efficient analysis of large cohorts. Polar metabolites were analyzed using the General Metabolics’ high-throughput, nontargeted metabolomics platform in negative ion acquisition mode. Technical replicates were acquired for all samples. The data obtained, covering a mass-to-charge ratio (*m/z*) range of 50–1,000 at a flow rate of 150 μl min^−1^, were subjected to further analysis through centroiding on a high-performance computing cluster using MATLAB R2018a. The resultant matrix was constructed from centroid lists. Annotation coverage was typical for human plasma, with 1,266 annotated ions and 1,339 unique molecular formulas matched against ion libraries derived from the HMDB (https://www.hmdb.ca/), Kyoto Encyclopedia of Genes and Genomes (KEGG) (https://www.genome.jp/kegg/) and Chemical Entities of Biological Interest (ChEBI) (https://www.ebi.ac.uk/chebi/) databases within 0.001 atomic mass unit of theoretical mass. The analytical method and approach to data processing and annotation have been described previously^57^. Given that the data were derived from human plasma, we prioritized annotation using the HMDB and converted KEGG or ChEBI IDs to HMDB IDs when available, using the Metabolite ID Conversion (https://www.metaboanalyst.ca/MetaboAnalyst/upload/ConvertView.xhtml, accessed March 2024). This resulted in a total of 935 metabolites that are present in the HMDB. Drugs that were detected using manual curating in the downstream analysis were flagged in the summary table and were disregarded in the analysis. Ion intensity values were normalized through log_2_; scaled values were used for the calculation of the PCA using the prcomp function (stats, v.3.6.2). Differential analysis for upregulated and downregulated metabolites between study time points was conducted using an LMM, including time point as a main fixed variable, age, BMI and physical activity level at baseline as covariates, and the individual as a random effect using the variancePartition package dream function followed by eBayes and topTable functions to extract the summary statics (v.1.24). Paired fold change values were calculated using the fcrosFCmat function from the fcros package (v.1.6.1), calculating the fold changes as a trimmed mean of the fold changes obtained using pairs of samples. DAMs were enriched for and assigned to chemical classes and enriched for metabolic pathways by querying the RaMP database (v.2.3.0, last updated 27 July 2023, accessed August 2024) using the website’s Application Programming Interface /api/combined-fisher-test and /api/combined-fisher-test using the R json function (jsonlite, v.1.8.4).

### Whole-blood transcriptome

Whole blood was collected in Qiagen PAXgene tubes. Total RNA was isolated using the QIAGEN PAXgene RNA isolation kit according to the manufacturer’s guidelines; eluted RNA was dissolved in RNase-free water. RNA quality was evaluated in the TapeStation 4200 (Agilent Technologies) by estimating 28S and 18S band integrity. TruSeq Stranded Total RNA with Ribo-Zero Globin (Illumina) was used to convert total RNA into double-stranded complementary DNA molecule libraries. Briefly, depletion of ribosomal and globin mRNA was performed on 750 ng purified total RNA using biotinylated, target-specific oligonucleotides combined with Ribo-Zero rRNA removal beads. Under elevated temperature, the remaining RNA was fragmented using divalent cations. The first strand was generated using SuperScript2 RT (Invitrogen) supplemented with actinomycin D, followed by second-strand synthesis with deoxyuridine triphosphate replacing deoxythymidine triphosphate. Before PCR amplification, adenylation of the 3′ ends and index adapter ligation were done before subsequent yielding of the final library. The remaining overhangs were converted into blunt ends via exonuclease and polymerase activities, and enzymes were removed. Selective DNA fragment enrichment with ligated adapter molecules was performed using Illumina PCR primers in 15 cycles of PCR reaction, followed by purification of complementary DNA using SPRI Beads (Beckman Coulter). High-throughput sequencing was carried out with the Illumina NovaSeq 6000 Sequencing System on an S4 flow cell with v.1.5 chemistry (76-bp paired-end reads); data were converted into FASTQ files using bcl2fastq2 v.2.20.

### Gene expression profiling and bioinformatic analysis

Raw RNA-seq data were aligned using STAR (v.2.7.3a) and human reference transcriptome version HG38 to generate the gene counts table. Raw gene counts were normalized using DESeq2 (v.1.26.0) in R (v.4.1.2)^58^. Each one of the dietary arms was analyzed separately, together with its control group; the following analysis was applied: low-expressed gene counts (genes with fewer than five counts in more than 10% of the samples) as well as hemoglobin or ribosomal genes were excluded, resulting in 19,887–20,585 genes present in each of the dietary groups and comparisons. Variance stabilizing transformation (VST) was applied on normalized data (exact numbers used for the analysis are provided in Supplementary Table 4). Before differential analysis, quality control was done with all study samples using robust PCA on VST-transformed corrected counts of all present genes using the PcaHubert function from the rrcov package (v.1.7-5)^59^, excluding six samples that presented high score and orthogonal distance. PCA was conducted using the prcomp function (stats, v.3.6.2) on scaled VST values or scaled VST values corrected for participants (removeBatchEffect function, limma package v.3.50.3). For the differential analysis, we followed the strategy for group-specific condition effects, with individuals nested within groups, which assumes a common time effect in the diet or control groups. The DESeq2 model design applied included the individual (ind.*n*), treatment (diet or control) and time point (baseline, after the intervention or at the follow-up) resulting in the following model: ~treatment + treatment:ind.*n* + treatment:time point. Differential expression analysis was performed between each two time points using the FDR correction method for *P*_adj_IHW^60^, setting the threshold for *P*_adj_ at 0.2, with no fold change threshold. Gene set enrichment analysis for the GO terms of DEGs was performed using the clusterProfiler package (v.4.2.2) using the compareCluster function with *P*_adj_ < 0.05 (with Benjamini–Hochberg correction) defining all expressed genes as the universe. The function simplify was used to reduce the number of similar terms (clusterProfiler), setting the threshold to 0.7; further selection of pathways of interest was done manually for visualization using dotplot or heatplot with the respective clusterProfiler visualization functions. The full list of DEGs and enriched GO terms is found in Supplementary Tables 14–19.

Transcription factor enrichment analysis was performed using the ChIP-X Enrichment Analysis 3 (ChEA3) tool via its Application Programming Interface (https://maayanlab.cloud/chea3/api/enrich/; accessed April 2024), using the jsonlite package in R (v.1.8.4)^61^. Transcription factor and gene targets found in the same DEG group were visualized in a circular plot using igraph (v.1.2.11) (Supplementary Table 19).

### Statistical analyses and graphics

All statistical analyses and visualizations were conducted in R (v.4.1.1.), using ggplot2 (v.3.3.5) unless otherwise specified. For all statistical analyses, intra-individual measurements were assessed using an LMM, with time point as the main fixed variable, and age, BMI and activity ranking at baseline as covariates. The individual was included as a random effect, using the dream function from the variancePartition package, followed by the eBayes and topTable functions (v.1.24) to extract summary statistics, or by including the individual in the model (see the DESeq analysis above). Measurements from the control groups were used for qualitative comparison with the intervention groups or as a contrast group for modeling transcriptional differences.

Paired fold change values were calculated using the fcrosFCmat function from the fcros package (v.1.6.1), calculating the fold changes as a trimmed mean of the fold changes obtained using pairs of samples. For gene expression, fold change values were calcualted using results function (see the DESeq analysis above). To reduce the burden of multiple comparisons in the omics data, differential plasma protein (Olink assay) and metabolite analysis was performed on analytes where time points contributed more than 5% of the variance. *P* values were calculated using two-tailed hypothesis testing, and Benjamini–Hochberg correction adjusting for multiple testing on the overall tests performed at each time point contrast to control the FDR, with a significance threshold set at *P*_adj_ < 0.05 when using mixed model analysis (setting the correction to ‘BH’, topTable function, variancePartition package). For transcriptome analysis, IHW was applied by setting the filter to ihw within the results function of the DESeq2 package, as described in the previous section.

### Ethics and inclusion statement

Researchers from KCMC University in Moshi, Tanzania, had an integral role throughout the research process, including study design and implementation, data ownership, intellectual property rights, and authorship of publications. The local relevance of the research was confirmed through collaboration with KCMC University partners. Roles and responsibilities were clearly defined among collaborators at the outset, and plans for capacity building of local researchers were discussed. Furthermore, local and regional research relevant to the study was carefully considered and appropriately cited. Lastly, benefit-sharing measures were discussed in collaboration with KCMC University researchers. The study was registered in the ISRCTN registry (ISRCTN15619939) before the start of the study. Full details of the trial protocol can be found in the Supplementary Information.

### Reporting summary

Further information on research design is available in the Nature Portfolio Reporting Summary linked to this article.

## Online content

Any methods, additional references, Nature Portfolio reporting summaries, source data, extended data, supplementary information, acknowledgements, peer review information; details of author contributions and competing interests; and statements of data and code availability are available at 10.1038/s41591-025-03602-0.

## Supplementary information

Supplementary Information Supplementary Figs. 1–5.

Reporting Summary

Supplementary Tables 1–19 Statistical and relevant analysis outputs.

## Figures and Tables

**Extended Data Table 1:**
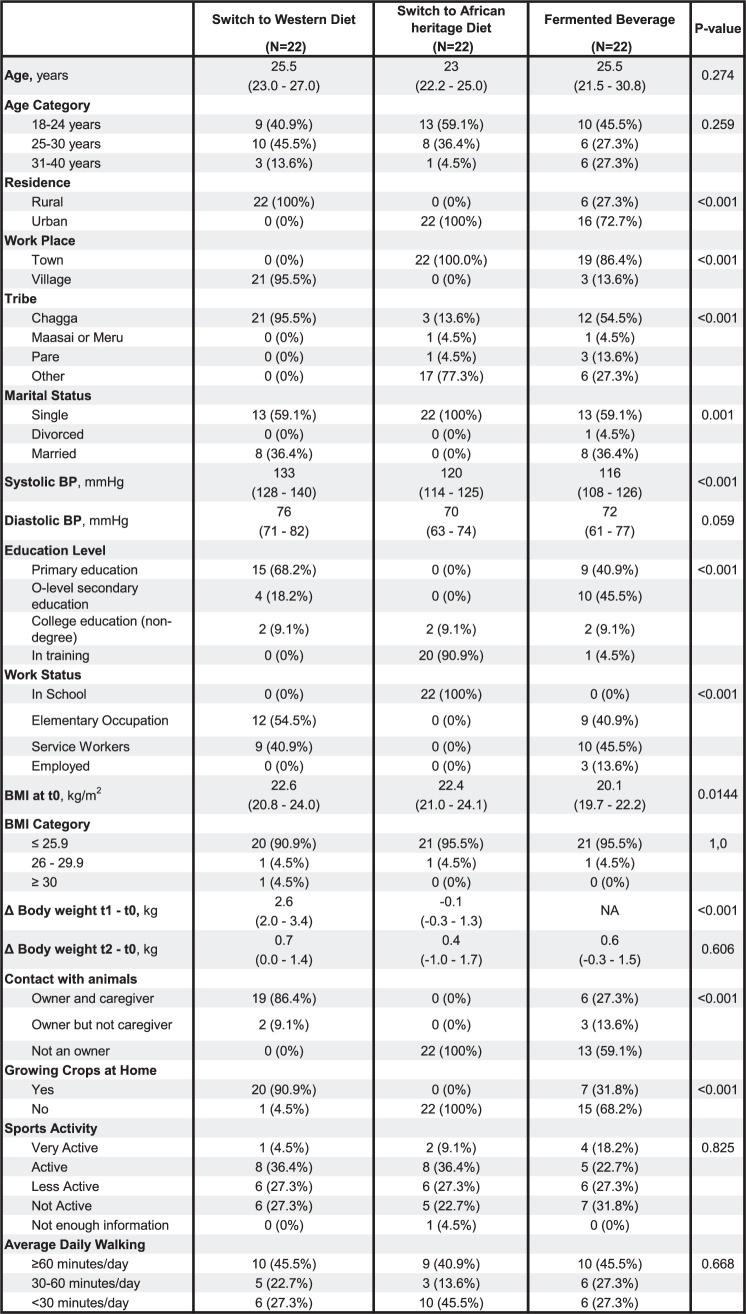
Demographic characteristics of the study participants

For continuous variables, data are presented as the median and interquartile range (IQR). For categorical variables, data are presented as counts with percentages in parentheses. A two-sided Kruskal–Wallis test was used to compare continuous variables, while a two-sided Fisher’s exact test was applied for categorical variables. One participant in the switch to WD group had incomplete metadata. Abbreviations: BMI, body mass index; BP, blood pressure.

**Extended Data Table 2:**
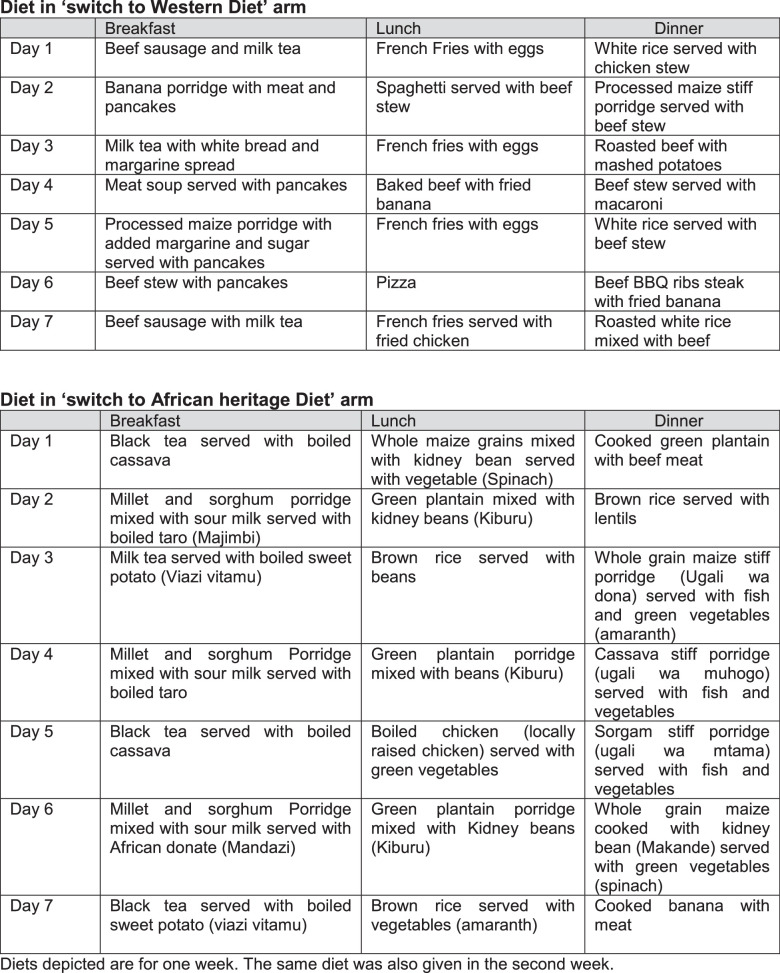
Dietary intervention menus

## Data Availability

All sequencing data have been deposited in the European Genome–phenome Archive under accession no. EGAS50000000317, hosted by the European Bioinformatics Institute and the Centre for Genomic Regulation. All additional datasets, including anonymized participant meta-data, nutritional information, circulating leukocyte data, whole-blood cytokine responses, and plasma proteome and metabolome data, are deposited in the Radboud Data Repository (https://data.ru.nl). Access to these datasets will be evaluated by a Data Access Committee to ensure compliance with the Tanzanian regulatory framework for data sharing (https://www.costech.or.tz/Files/Documents/1728287181.pdf). Researchers wishing to access the data should contact the corresponding authors Q.D.M. (quirijn.demast@radboudumc.nl) or G.T. (gtemba@kcmuco.ac.tz). Requests will be reviewed by the Data Access Committee and a decision will be provided within 4 weeks. Other databases used for the analysis include the dataset sourced from the Human Protein Atlas (https://www.proteinatlas.org/; https://www.proteinatlas.org/download/proteinatlas.tsv.zip) for the plasma proteome; the KEGG COMPOUND Database (https://www.genome.jp/kegg/compound/); the HMDB (https://www.hmdb.ca/); the ChEBI (https://www.ebi.ac.uk/chebi/); Metabolite ID Conversion (https://www.metaboanalyst.ca/MetaboAnalyst/upload/ConvertView.xhtml); the RaMP database (https://rampdb.nih.gov/) for the metabolome analysis; and GO (https://geneontology.org/) and ChEA3 (https://maayanlab.cloud/chea3/api/enrich/) for the transcriptome analysis.

## Code availability

All code scripts used for the analysis of the data are available at https://github.com/TalPecht/dietstudy_Temba_Pecht_2024/.
