# Supplementary material for: Immune and metabolic effects of African heritage diets versus Western diets in men: a randomized controlled trial
Source: Nat Med. 2025 Apr 3;31(5):1698–711. doi: 10.1038/s41591-025-03602-0 (PMC12092257; doi:10.1038/s41591-025-03602-0)
Supplement: Supplementary file 1 — Supplementary Figs. 1–5. [file 41591_2025_3602_MOESM1_ESM.pdf]

nature medicine

Supplementary information

https://doi.org/10.1038/s41591-025-03602-0

Immune and metabolic effects of African heritage diets versus Western diets in men: a randomized controlled trial

In the format provided by the authors and unedited

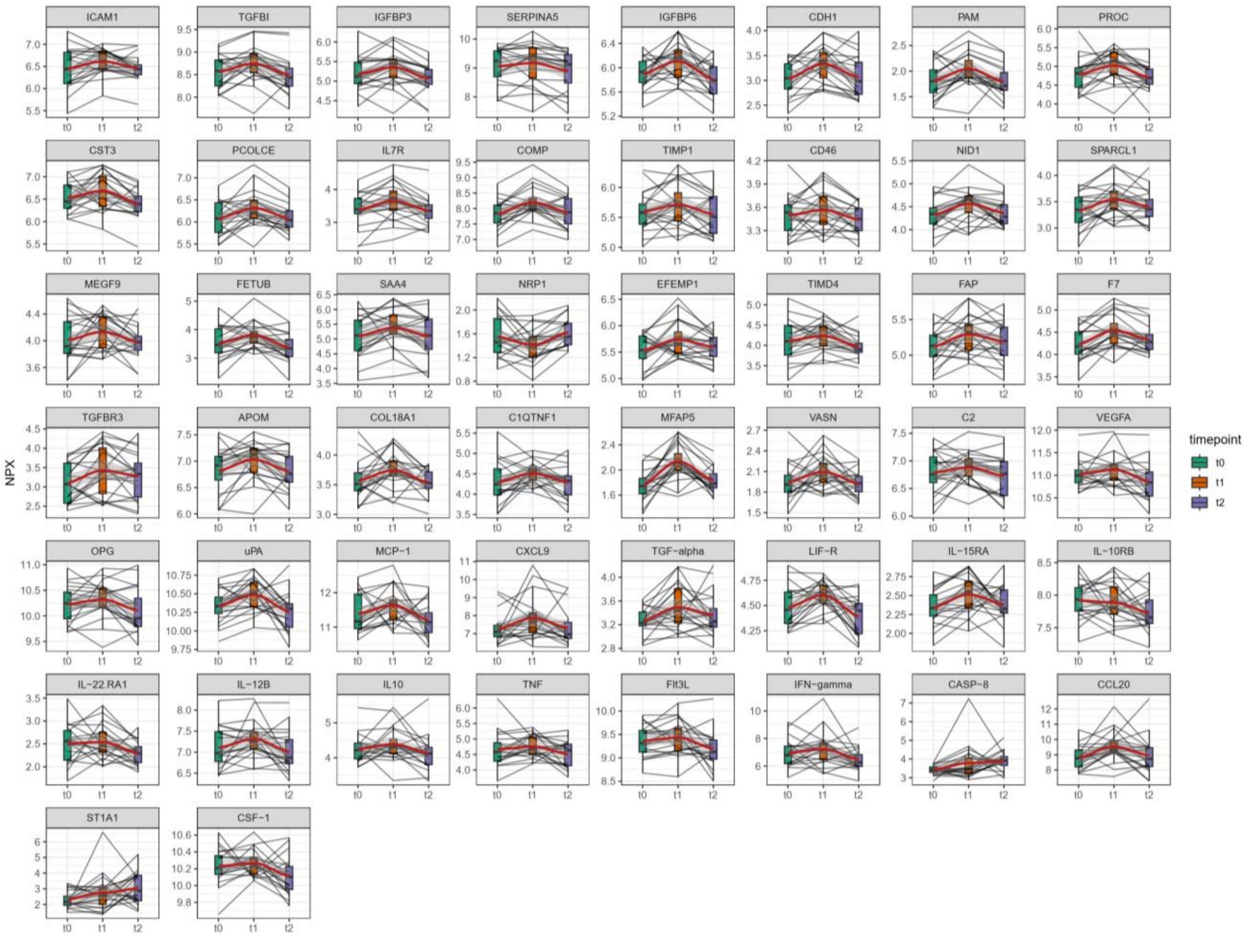

Supplementary Figure 1. Changes in plasma proteome in Western Diet (WD) intervention group. Targeted plasma proteomics was conducted using the inflammatory and cardiometabolic panel (Olink® Proteomics AB). Samples from N=20 individuals in the Western Diet (WD) intervention arm were obtained at baseline (t0), post-intervention (t1, week 2), and at four weeks follow-up (t2). Statistically significant differentially abundant proteins (DAPs, linear mixed model including time point, BMI, age and physical activity level as fixed variables and participant ID as a random effect with two-sided hypothesis testing, Benjamini Hochberg adjusted *P* value <0.05) are presented in combined box- and line- plots with group-trend lines indicating levels of measured parameters at the 3 timepoints. Proteins levels are presented as NPX values. Presented here are the measures that are not included in the main figure.

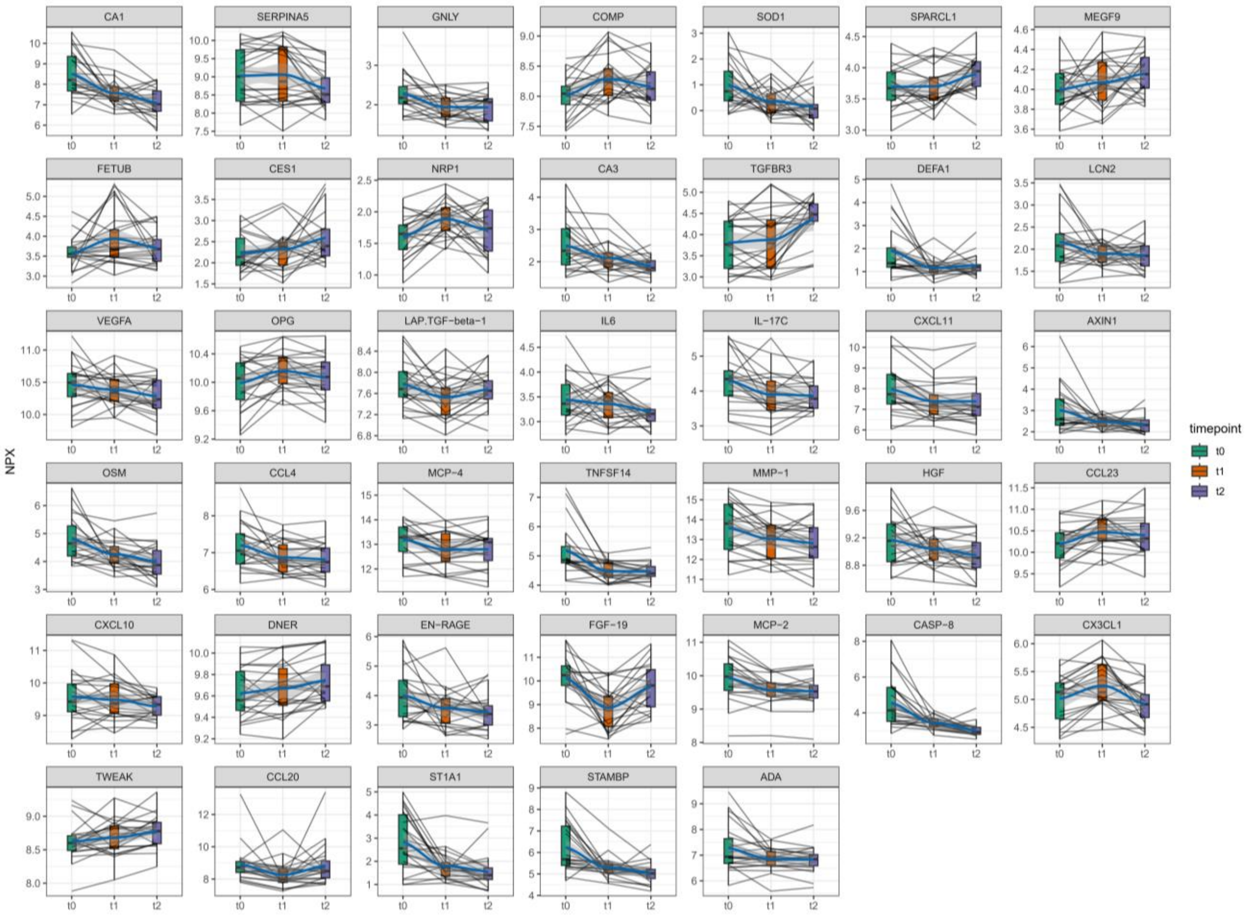

Supplementary Figure 2. Changes in plasma proteome in switch to African heritage Diet group. Targeted plasma proteomics was conducted using the inflammatory and cardiometabolic panel (Olink® Proteomics AB). Samples from N=22 individuals in the were obtained at baseline (t0), post-intervention (t1, week 2), and at four weeks follow-up (t2). Statistically significant differentially abundant proteins (DAPs, linear mixed model including time point, BMI, age and physical activity level as fixed variables and participant ID as a random effect with two-sided hypothesis testing, Benjamini Hochberg adjusted *P* value <0.05) are presented in combined box- and line-plots with group-trend lines indicating levels of measured parameters at the 3 timepoints. Proteins levels are presented as NPX values. Presented here are the measures that are not included in the main figure.

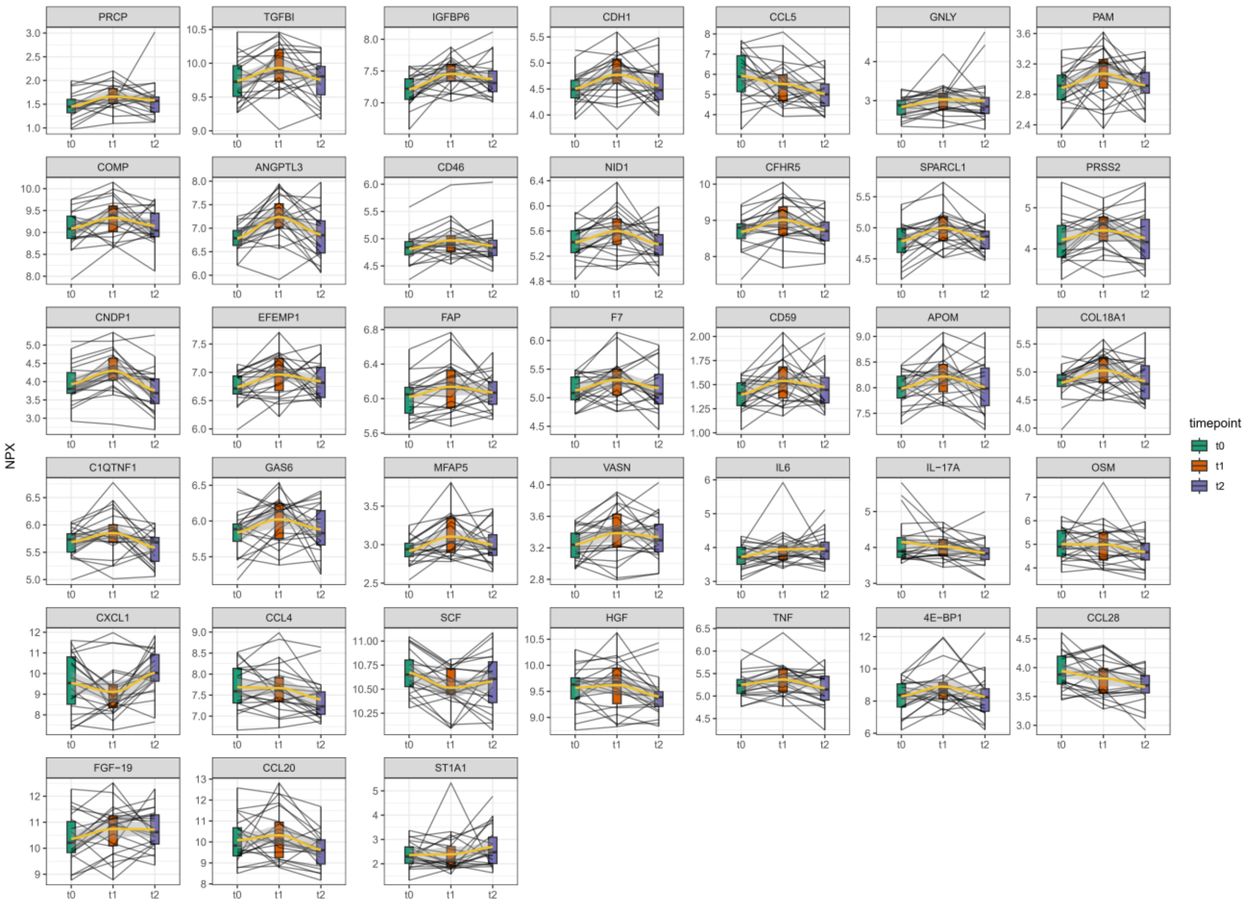

Supplementary Figure 3. Changes in plasma proteome in fermented beverage intervention group. Targeted plasma proteomics was conducted using the inflammatory and cardiometabolic panel (Olink® Proteomics AB). Samples from N=22 individuals in the fermented beverage intervention arm were obtained at baseline (t0), post-intervention (t1, week 1), and at four weeks follow-up (t2). Statistically significant differentially abundant proteins (DAPs, linear mixed model including time point, BMI, age and physical activity level as fixed variables and participant ID as a random effect with two-sided hypothesis testing, Benjamini Hochberg adjusted *P* value <0.05) are presented in combined box- and line- plots with group-trend lines indicating levels of measured parameters at the 3 timepoints. Proteins levels are presented as NPX values. Presented here are the measures that are not included in the main figure.

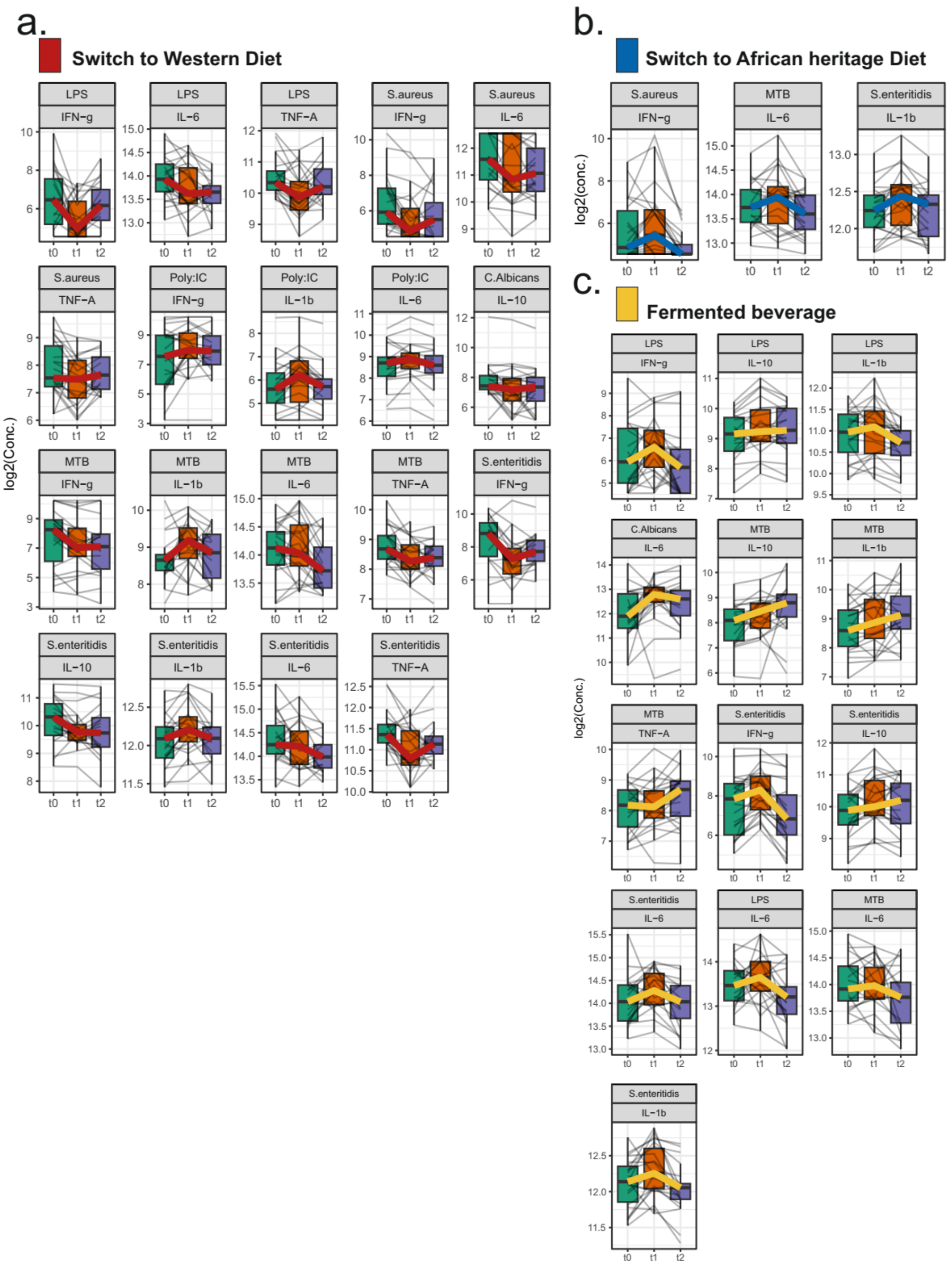

Supplementary Figure 4. Changes in whole blood cytokine production to stimulation with various microbial pathogens. Whole blood obtained at baseline (t0), post-intervention (t1, week 2 for the dietary intervention and one week for the fermented beverage intervention), and four weeks follow-up (t2), was subjected to *ex-vivo* stimulation for 48 hours using *Candida albicans* hyphae (1x10^{7}/mL), *E. coli* lipopolysaccharide (LPS; 10ng/mL), *Mycobacterium tuberculosis* (MTB*;* 5µg/mL), *Salmonella enteritidis* (S. enteritidis;1x10^{6}/mL), *Staphylococcus aureus* (S. aureus; 1x10^{6}/mL) and the TLR3 agonist Poly(I:C) (50µg/mL). Cytokine concentrations were determined in the supernatants of the assays done at the three timepoints from N=19, 21, 22 individuals in the switch to Western Diet (WD), Traditional Diet (TD) or fermented beverage groups. **a, b, c -** Combined box- and line- plots with group-trend line indicating levels of measured parameters at the 3 timepoints. Lines are grouped per-participant. Cytokine production is presented as log$_2$ concentrations of IFN-γ, IL-10, IL-1β, IL-6, and TNF in the supernatant.

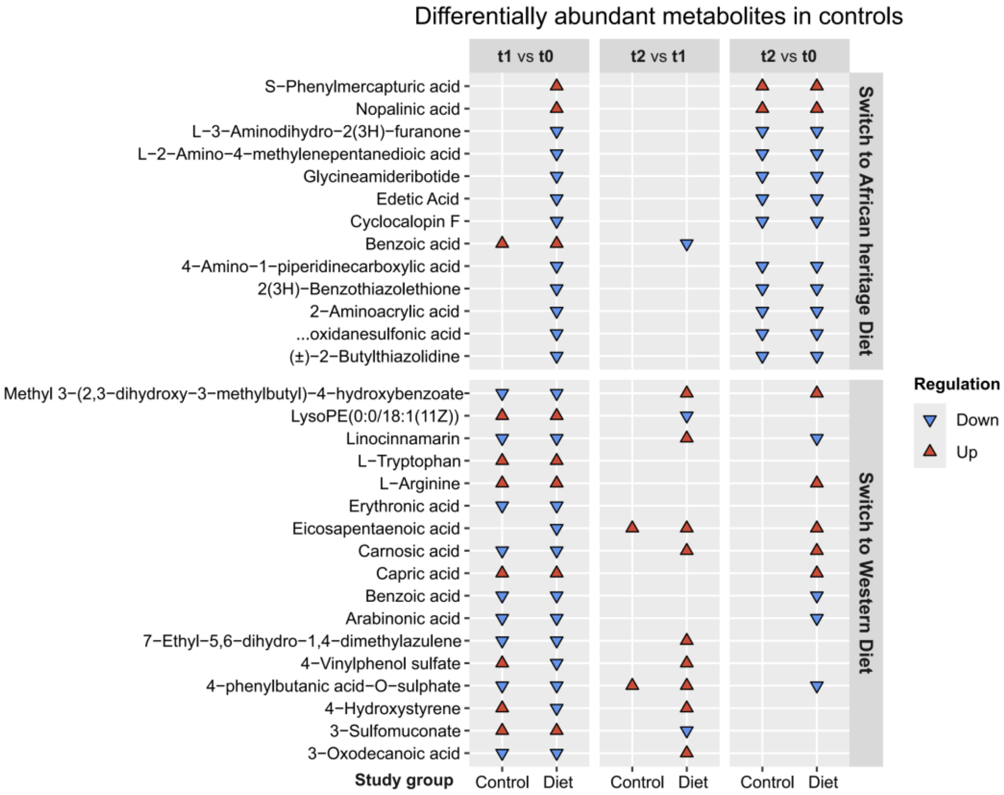

Supplementary Figure 5. Differentially abundant metabolites (DAM) in the controls. Triangle plot presenting the differentially abundant metabolites (DAM, y-axis) present in the controls in the switch to Western diet (lower panel) and switch to African heritage Diet (upper panel) groups (N=5 in each control group) at each time-point comparison in the control or diet study groups (x-axis). Color represents regulation direction, with upregulated metabolites in red and downregulated metabolites in blue).
