# Supplementary material for: Immune and metabolic effects of African heritage diets versus Western diets in men: a randomized controlled trial
Source: Nat Med. 2025 Apr 3;31(5):1698–711. doi: 10.1038/s41591-025-03602-0 (PMC12092257; doi:10.1038/s41591-025-03602-0)
Supplement: Supplementary file 2 — Reporting Summary [file 41591_2025_3602_MOESM2_ESM.pdf]

nature portfolio

Corresponding author(s): Dr. Quirijn de Mast

Last updated by author(s): Jan 31, 2025

Reporting Summary

Nature Portfolio wishes to improve the reproducibility of the work that we publish. This form provides structure for consistency and transparency in reporting. For further information on Nature Portfolio policies, see our Editorial Policies and the Editorial Policy Checklist.

Statistics

For all statistical analyses, confirm that the following items are present in the figure legend, table legend, main text, or Methods section.

n/a	Confirmed	
☐	☒	The exact sample size (*n*) for each experimental group/condition, given as a discrete number and unit of measurement
☐	☒	A statement on whether measurements were taken from distinct samples or whether the same sample was measured repeatedly
☐	☒	The statistical test(s) used AND whether they are one- or two-sided<br>*Only common tests should be described solely by name; describe more complex techniques in the Methods section.*
☐	☒	A description of all covariates tested
☐	☒	A description of any assumptions or corrections, such as tests of normality and adjustment for multiple comparisons
☐	☒	A full description of the statistical parameters including central tendency (e.g. means) or other basic estimates (e.g. regression coefficient) AND variation (e.g. standard deviation) or associated estimates of uncertainty (e.g. confidence intervals)
☐	☒	For null hypothesis testing, the test statistic (e.g. *F*, *t*, *r*) with confidence intervals, effect sizes, degrees of freedom and *P* value noted<br>*Give P values as exact values whenever suitable.*
☒	☐	For Bayesian analysis, information on the choice of priors and Markov chain Monte Carlo settings
☐	☒	For hierarchical and complex designs, identification of the appropriate level for tests and full reporting of outcomes
☐	☒	Estimates of effect sizes (e.g. Cohen's *d*, Pearson's *r*), indicating how they were calculated

Our web collection on statistics for biologists contains articles on many of the points above.

Software and code

Policy information about availability of computer code

Data collection	Data were collected using cloud based electronic data capture (CASTOR EDC 2021.1 EU server)
Data analysis	All statistical analyses and visualizations were conducted in R (version 4.1.1.), mostly using ggplot2 (version 3.3.5) , clusterProfiler (version 4.2.2) for transcriptome enrichment heatmaps, and circlize (version 0.4.14) for transcription factors and gene targets circular plots . For all statistical analyses, the intra-individual measurements were assessed using a linear mixed model, with time-point as the main fixed variable, and age, BMI and activity ranking at baseline as covariates . The individual was included as random effect, using the dream function, followed by the eBayes and topTable functions to extract summary statistics (variancePartition package, version 1.24), paired analysis for the individual or by including the individual in the model (DESeq2 version 1.26.0). Measurements from the control groups were used for qualitative comparison with the intervention groups or as a contrast group for modeling transcriptional differences. Paired fold-change values were calculated using the function fcrosFCmat from the package fcros (version 1.6.1) calculating the fold changes as a trimmed mean of the fold changes obtained using pairs of samples. To reduce the burden of multiple comparisons in the omics data, differential plasma protein (Olink® assay) and metabolite analysis was was performed on proteins where time points contributed more than 5% of the variance ((variancePartition package, version 1.24). Statistical significance was determined using the false discovery rate (FDR) method, adjusting for multiple testing across all tests performed in each time-point contrast using the Benjamini-Hochberg (BH) procedure when utilizing mixed models analysis (setting the correction argument to “BH”, topTable function, variancePartition package). For DESeq differential analysis of the transcriptome BH procedure was used along with Independent Hypothesis Weighting (setting the filter argument to ihw, results function, DESeq2 package). Two-sided hypothesis testing was performed, with the significance level set at an adjusted p-value of <0.05 for all differential analyses, except for RNA-seq, where it was set to 0.2. Genes overrepresentation (enrichment) analysis for Gene Ontology terms was done on DE genes using the clusterProfiler package compareCluster function (version 4.2.2). Transcription factor (TF) enrichment analysis of the DE genes was performed using the ChEA3 (ChIP-X Enrichment Analysis 3) tool via its API: https://maayanlab.cloud/chea3/api/enrich/ (accessed on April 2024) using R json utility (jsonlite, version 1.8.4). Fisher'r over-represenation analysis for metbolic pathways was done using

Metabolomic Pathways (RaMP-DB) website's Application Programming Interface (API, accessed on July 2024) "/api/combined-fisher-test" and "/api/combined-fisher-test" using R json utility (jsonlite, version 1.8.4). Code analysis scripts are available at https://github.com/TalPecht/dietstudy_Temba_Pecht_2024.

For manuscripts utilizing custom algorithms or software that are central to the research but not yet described in published literature, software must be made available to editors and reviewers. We strongly encourage code deposition in a community repository (e.g. GitHub). See the Nature Portfolio guidelines for submitting code & software for further information.

Data

Policy information about availability of data

All manuscripts must include a data availability statement. This statement should provide the following information, where applicable:

- Accession codes, unique identifiers, or web links for publicly available datasets
- A description of any restrictions on data availability
- For clinical datasets or third party data, please ensure that the statement adheres to our policy

All sequencing data have been deposited in the European Genome–phenome Archive (EGA) under accession code EGAS50000000317 (https://ega-archive.org/studies/EGAS50000000317), hosted by the European Bioinformatics Institute (EBI) and the Centre for Genomic Regulation (CRG). All additional datasets—including anonymized participant metadata, nutritional information, circulating leukocyte data, whole blood cytokine responses, plasma proteome and metabolome data produced in this study, as well as the data from the 300 TZFG cohort —are deposited in the Radboud Data Repository (https://data.ru.nl). Access to these datasets will be evaluated by a Data Access Committee (DAC) to ensure compliance with the Tanzanian regulatory framework for data sharing (https://www.costech.or.tz/Files/Documents/1728287181.pdf). Researchers wishing to access the data should contact the corresponding author, Quirijn de Mast (quirijn.demast@radboudumc.nl) or the author Godfrey Temba (gtemba@kcmuco.ac.tz). Requests will be reviewed by the DAC, and a decision will be provided within four weeks. Other databases used for analysis include: The Human Protein Atlas (https://www.proteinatlas.org/download/proteinatlas.tsv.zip) for plasma proteom; KEGG (https://www.genome.jp/kegg/compound/), HMDB (https://www.hmdb.ca/), ChEBI (https://www.ebi.ac.uk/chebi/), MetaboAnalyst ID converter (https://www.metaboanalyst.ca/MetaboAnalyst/upload/ConvertView.xhtml) and RampDB (https://rampdb.nih.gov/) for metabolome analysis; GO (https://geneontology.org/) for plasma metabolome; GO (https://geneontology.org/) and ChEA3 (https://maayanlab.cloud/chea3/api/enrich/) for transcriptome analysis.

Human research participants

Policy information about studies involving human research participants and Sex and Gender in Research.

Reporting on sex and gender

Only men were enrolled. This is addressed in the manuscript.

Population characteristics

Healthy male volunteers (n=77) from Kilimanjaro region in Northern Tanzania were recruited: (a) males residing in the rural village Uru Shimbwe Juu or its environs, adhering to a predominantly traditional Tanzanian diet, and (b) males residing in Moshi town, adhering to a predominantly Western-style diet.
Only participants adhering to either a traditional Kilimanjaro-style or Western-style diet were further considered for participation. Other inclusion criteria were age 20-40 years, BMI between 18 and 25 kg/m2, residing in either the rural or urban areas of Moshi for a minimum of one month preceding the study, and commitment to stay in the study area throughout the intervention period. Exclusion criteria were a positive result of HIV or malaria rapid test, notable blood pressure abnormalities, elevated fasting blood sugar level, food allergies, recent acute illness, use of medication, antibiotics or vaccination within the last three months, previous hospitalization within the past year, presence of chronic conditions such as active malignancy, liver or kidney disease, tuberculosis, chronic hepatitis B or C infection, or unwillingness to consume alcohol.

Recruitment

Information about the study was disseminated through leaflets, community talks, and announcements during church gatherings. We conducted a dietary recall questionnaire over three non-consecutive 24-hour periods within one week, including one weekend day, to assess the dietary habits of eligible volunteers who agreed to participate. The questionnaire captured details on different foods, as well as estimated portion sizes (small, medium, or large) and preparation methods for each food item. A nutritionist evaluated the questionnaires to confirm that participants were following either a Kilimanjaro heritage-style diet or a Western-style diet. This assessment relied primarily on the nutritionist's expertise and knowledge of local dietary habits rather than a standardized set of criteria, incorporating traditional understanding of food patterns.

Several potential biases may have influenced the study outcomes. The exclusive recruitment of young, healthy men inherently limits the generalizability of the findings. Additionally, self-selection bias may have occurred, as individuals with a strong interest in health, nutrition, or research may have been more likely to participate. Conversely, the provision of free meals may have drawn participants primarily motivated by financial incentives rather than genuine interest in the dietary intervention. This mix of motivations could have impacted both participant engagement and the overall composition of the study sample.

Ethics oversight

The current study was approved by the Ethical Committees of the Kilimanjaro Christian Medical University College (CREC) (No. 2483) and the National Institute for Medical Research in Tanzania (No. NIMR/HQ/R.8a/Vol.IX 3570).

Note that full information on the approval of the study protocol must also be provided in the manuscript.

Field-specific reporting

Please select the one below that is the best fit for your research. If you are not sure, read the appropriate sections before making your selection.

☒ Life sciences ☐ Behavioural & social sciences ☐ Ecological, evolutionary & environmental sciences

For a reference copy of the document with all sections, see nature.com/documents/nr-reporting-summary-flat.pdf

Life sciences study design

All studies must disclose on these points even when the disclosure is negative.

Sample size	The sample size for this study was determined based on functional immune response outcomes, specifically estimated whole blood cytokine (TNF) production, as observed in urban and rural populations in a previous cross-sectional study (Temba et al. Nature Immunology 2020).
Data exclusions	One participant in the arm switching from an African heritage to a Western diet withdrew from further participation following the baseline visit and was excluded from all analyses. In addition, two participants in this arm who completed the dietary intervention had no blood sample taken at the follow-up time point (t2), resulting in N=22, N=22 and N=20 at the respective ime points: baseline (t0), post-intervention (t1) and follow up (t2), respectively.<br>Specifically for RNAseq analysis: Prior to differential analysis, quality control was done with all study samples using robust PCA on VST-transformed corrected counts of all present genes using the PcaHubert function from the rrcov package (version 1.7-5), which resulted in exclusion 6 samples that presented high score and orthogonal distance.
Replication	We replicated the changes in the plasma proteome using data from the 300 Tanzania Functional Genomics (TZFG) cohort, a cross-sectional study of urban or rural-living Tanzanians (n = 295) from the same region. Participants were previously categorized into “Kilimanjaro heritage-style diet” (n = 138) and “Western-style diet” clusters based on food-derived plasma metabolome profiles (Extended Data Fig. 6a). Differential analysis using the Olink inflammation panel identified 18 proteins that were lower in the heritage-style diet cluster compared to the Western-style diet cluster (Supplementary Table S19). Among these, 83% (15 proteins) also decreased in the current Western to heritage diet arm (Extended Data Fig. 6b,c; Supplementary Table S19). These proteins included chemokines CXCL1, CXCL5, CXCL6, CXCL11, MCP-2, and MCP-4.
Randomization	Participants in the dietary intervention arms were randomized to either the dietary intervention (n=23 in the heritage to Western diet arm; n=22 in Western to heritage diet arm) or remaining on the habitual diet (n=5 in each arm, ‘controls’). No controls were included in the fermented beverage arm. The randomization was conducted by an independent statistician using a lottery method. Each pre-selected participant was assigned a number based on the dietary assessment, from which five numbers (controls) were randomly selected.
Blinding	Investigators were not blinded during data collection or analysis. Blinding was deemed unnecessary due to the open-label design of the dietary intervention study, where the nature of the intervention made blinding impractical and unlikely to influence the objective outcomes being measured

Reporting for specific materials, systems and methods

We require information from authors about some types of materials, experimental systems and methods used in many studies. Here, indicate whether each material, system or method listed is relevant to your study. If you are not sure if a list item applies to your research, read the appropriate section before selecting a response.

Materials & experimental systems

n/a	Involved in the study	
☒	☐	Antibodies
☒	☐	Eukaryotic cell lines
☒	☐	Palaeontology and archaeology
☒	☐	Animals and other organisms
☐	☒	Clinical data
☒	☐	Dual use research of concern

Methods

n/a	Involved in the study	
☒	☐	ChIP-seq
☒	☐	Flow cytometry
☒	☐	MRI-based neuroimaging

Clinical data

Policy information about clinical studies

All manuscripts should comply with the ICMJE guidelines for publication of clinical research and a completed CONSORT checklist must be included with all submissions.

Clinical trial registration	ISRCTN15619939
Study protocol	https://doi.org/10.1186/ISRCTN15619939
Data collection	This study was conducted in Moshi district in the Kilimanjaro region of northeastern Tanzania between April to August 2021, which

Data collection

coincides largely with the wet season. Moshi town serves as the administrative, commercial, and educational center for the region, with a population of approximately 220,000 inhabitants. Moshi town borders the foothills of the southern slopes of Mount Kilimanjaro. These foothills are rural areas where people live in smaller villages and are engaged in subsistence agriculture and animal husbandry. Most of the people living in the foothills belong to the Chagga tribe. For this study, the rural dwellers were mainly recruited from the village Uru Shimbwe Juu, which is located approximately 15 km from Moshi town at an elevation of 1,978 meters.

Outcomes

Primary outcomes:
-Circulating inflammation-related human protein biomarkers at baseline, post-intervention and 4 weeks post intervention, measured using the Olink® Targeted Proteomics platform, which utilizes Proximity Extension Assay (PEA) technology
-The capacity of the circulating immune cells to produce inflammatory cytokines in ex vivo whole blood stimulation to different stimuli, measured using ELISA on the culture supernatant ,at baseline (day 0), post intervention and 4 weeks post-intervention.
-Whole blood transcriptome measured using RNAseq technology with NovaSeqTM Sequencing System at baseline, post-intervention and 4 weeks later
- Plasma metabolome assessed using high-throughput mass spectrometry (untargeted metabolomics) at baseline (day 0), post intervention and at 4 weeks post-intervention

Secondary outcome
- Gut microbiome composition measured using metagenomic sequencing of stool samples collected at baseline, post intervention and 4 weeks post-intervention
